# Epigenetic memory *via* concordant DNA methylation is inversely correlated to developmental potential of mammalian cells

**DOI:** 10.1371/journal.pgen.1007060

**Published:** 2017-11-06

**Authors:** Minseung Choi, Diane P. Genereux, Jamie Goodson, Haneen Al-Azzawi, Shannon Q. Allain, Noah Simon, Stan Palasek, Carol B. Ware, Chris Cavanaugh, Daniel G. Miller, Winslow C. Johnson, Kevin D. Sinclair, Reinhard Stöger, Charles D. Laird

**Affiliations:** 1 Department of Biology, University of Washington, Seattle, Washington, United States of America; 2 Department of Computer Science, Princeton University, Princeton, New Jersey, United States of America; 3 Broad Institute of Massachusetts Institute of Technology and Harvard University, Cambridge, Massachusetts, United States of America; 4 Department of Pathology, University of Washington School of Medicine, Seattle, Washington, United States of America; 5 School of Biosciences, University of Nottingham, Sutton Bonington Campus, Loughborough, Leicestershire, United Kingdom; 6 Department of Biostatistics, University of Washington, Seattle, Washington, United States of America; 7 Department of Mathematics, Princeton University, Princeton, New Jersey, United States of America; 8 Department of Comparative Medicine, University of Washington School of Medicine, Seattle, Washington, United States of America; 9 Institute for Stem Cell and Regenerative Medicine, University of Washington, Seattle, Washington, United States of America; 10 Department of Pediatrics, University of Washington School of Medicine, Seattle, Washington, United States of America; 11 Department of Biology, Carnegie Mellon University, Pittsburgh, Pennsylvania, United States of America; 12 Department of Genome Sciences, University of Washington, Seattle, Washington, United States of America; Stanford University School of Medicine, UNITED STATES

## Abstract

In storing and transmitting epigenetic information, organisms must balance the need to maintain information about past conditions with the capacity to respond to information in their current and future environments. Some of this information is encoded by DNA methylation, which can be transmitted with variable fidelity from parent to daughter strand. High fidelity confers strong pattern matching between the strands of individual DNA molecules and thus pattern stability over rounds of DNA replication; lower fidelity confers reduced pattern matching, and thus greater flexibility. Here, we present a new conceptual framework, Ratio of Concordance Preference (RCP), that uses double-stranded methylation data to quantify the flexibility and stability of the system that gave rise to a given set of patterns. We find that differentiated mammalian cells operate with high DNA methylation stability, consistent with earlier reports. Stem cells in culture and in embryos, in contrast, operate with reduced, albeit significant, methylation stability. We conclude that preference for concordant DNA methylation is a consistent mode of information transfer, and thus provides epigenetic stability across cell divisions, even in stem cells and those undergoing developmental transitions. Broader application of our RCP framework will permit comparison of epigenetic-information systems across cells, developmental stages, and organisms whose methylation machineries differ substantially or are not yet well understood.

## Author summary

As stem cells differentiate, they acquire epigenetic marks that activate some genes and silence others, eventually producing the profiles that define specific cell lineages. While existing approaches can reveal the differentiation state of a given cell or the activity state of a given gene, none can locate an individual genomic region along the epigenetic continuum between flexible and fixed. To address this challenge, we introduce a new framework to infer epigenetic stability from the concordance of DNA methylation patterns on the two strands of individual DNA molecules. For cells of all developmental potentials, we find that top- and bottom-strand methylation patterns match far more often than expected by chance alone. As cells differentiate, the fidelity of pattern transfer increases, thereby resulting in higher epigenetic stability; dedifferentiation is characterized by declining stability. Our metric has the potential to identify genomic regions that remain sensitive to environmental signals well beyond the interval of lineage specification.

## Introduction

Organismal development is characterized by a shift from the phenotypic flexibility of embryonic cells to the canalized identities of differentiated cells. To achieve stable gene-regulatory states in terminally differentiated cells, organisms ranging from Archaea to humans use a variety of epigenetic mechanisms, including DNA methylation. Perturbation of the state of DNA methylation at various loci in differentiated cells is associated with several human cancers [1–3]. In turn, restoring epigenetic flexibility of some loci has proven challenging during efforts to create induced pluripotent stem (iPS) cells [4]. Together, these findings highlight the importance of shifting ratios of epigenetic flexibility and stability in establishing cellular identity.

There exists an extensive literature documenting changes in single-locus and genome-wide methylation frequencies at various stages of development [5, 6]. Most genomic regions in primordial germ cells (PGCs), for example, are known to undergo dramatic and rapid shifts in DNA methylation frequency [7]. It is now clear that mammalian stem cells can utilize active demethylation [8], highlighting the potential for both gain and loss of cytosine methylation to impact the overall methylation frequency and, perhaps, stability of a given genomic region during development.

High concordance of methylation in differentiated cells, with matching states for parent and daughter DNA strands at individual CpG/CpG dyads, is considered to be a hallmark of conservative epigenetic processes [9–13]. For earlier stages of development, however, questions remain regarding the extent of concordance. For example, do methylation patterns in dividing embryonic stem cells arise entirely by random placement of methyl groups, or is concordance favored to some degree?

Recent work has begun to address these questions [7, 14–18]. Shipony *et al.* [16] analyzed DNA methylation patterns in populations of cultured cells established from single founder cells. Under this approach, the degree of stability was inferred from the extent of congruence among single-stranded patterns collected from cultured descendant cells. The observation of substantial pattern diversity among cells separated by many rounds of division led Shipony *et al.* [16] to conclude that the bulk of methylation in human embryonic stem (ES) and induced pluripotent stem (iPS) cells arises through “dynamic”—that is, non-conservative—DNA methylation processes rather than through the “static”—that is, conservative—processes that were emphasized in earlier studies [10, 11, 19]. Using data collected by hairpin-bisulfite PCR [13], which yields double-stranded DNA methylation patterns, other studies suggested that dynamic processes contribute substantially to DNA methylation in cultured mouse ES cells, but perhaps not to the exclusion of the conservative processes that dominate at many loci in adult differentiated cells [7, 14, 15, 17, 18].

To fully characterize the balance between conservative and non-conservative methylation processes, it is necessary to quantify the extent to which the arrangement of methylation in a given set of patterns deviates from the null assumption of random placement. To assess and visualize such deviations, we here introduce a new metric, Ratio of Concordance Preference (RCP), which utilizes double-stranded methylation data. Here, as previously, we use the term *double-stranded DNA methylation pattern* to refer to the overall pattern of methylation on both top and bottom strands of an individual double-stranded DNA molecule. Double-stranded patterns provide information on the extent of matching between methylation states on parent and daughter strands, which are separated by exactly one round of DNA replication. RCP requires no assumptions about the enzymatic mechanisms of methylation and demethylation, and so enables comparison across diverse species and developmental stages.

Jeltsch and Jurkowska [20] have emphasized the balance of methylating and demethylating processes—rather than the propagation of specific methylation patterns—as the primary determinant of the nature of the patterns present in a given cellular population at a given time. In this framework, RCP can be thought of as a metric for quantifying the extent to which the set of patterns produced by a given system of methylating and demethylating processes deviates from the set of patterns expected if methyl groups are placed entirely at random.

In parameterizing RCP, we use the term “conservative”, in lieu of “static” as used previously [16], to describe processes that preferentially establish concordant as opposed to discordant methylation states. We consider non-conservative processes, described previously as “dynamic” [16], as having one of two forms: “random” processes, which add or remove methyl groups with equal preference for concordance and for discordance, and “dispersive” processes, which preferentially establish discordant methylation states.

We validate our RCP framework by confirming its ability to identify systems in which contributions from conservative processes are nearly complete or nearly absent, as well as systems on the continuum between these extremes. We apply this new framework to our authenticated, double-stranded DNA methylation patterns, both published and previously unpublished, collected by dideoxy sequencing from DNA of human and murine cells. To expand the data available for this initial RCP analysis, we also examine double-stranded methylation patterns from three recent publications [14, 15, 17]. To improve our understanding of transitions between stem and differentiated cells, we ask: (*i*) how strong are preferences for concordant DNA methylation states in cultured stem cells?; (*ii*) do concordance preferences change as cultured cells shift between stem and differentiated states?; and (*iii*) in the developing embryo, do stem cells of various potencies have preferences that mirror those in cultured stem cells?

## Results and discussion

### Ratio of Concordance Preference is defined for all possible configurations of methylation at symmetric nucleotide motifs

We have developed the Ratio of Concordance Preference (RCP) to assess the strategy of binary information transfer, with focus on the degree to which exact information is conserved. We apply our RCP framework to DNA methylation in mammalian cells. Our goal is to infer whether and how much the system of processes that established a given set of methylation patterns prefers concordant to discordant methylation states. This general formulation is free of assumptions about the molecular mechanisms whereby methylation is added to and removed from DNA.

In our data from double-stranded DNA molecules from human and mouse, methylation occurs principally at the CpG motif. This symmetric motif may be written as CpG/CpG, which we here term “CpG dyad”. CpG dyads have opportunities for methylation on both strands. The methylation state of a dyad thus takes one of three forms: fully methylated, at frequency *M*, with methylated cytosines on both strands; hemimethylated, at frequency *H*, with a methylated cytosine on only one strand; and unmethylated, at frequency *U*, with neither cytosine methylated. The RCP framework can also be extended to non-CpG methylation at symmetric nucleotide motifs.

To infer concordance preference for sets of double-stranded methylation patterns, we use the overall frequency of methylation, *m*, and the frequency of unmethylated dyads, *U*, of each data set. Because *m* is derived from the three dyad frequencies, the pair (*m*, *U*) encompasses the full information available from the implicit dyad frequencies, *M* and *H*. RCP evaluates the extent of deviation from expectations under a random model in which the system has no preference for either concordant or discordant placement of methyl groups, and is defined as:

$$\begin{array}{c} \text{RCP}=\frac{\sqrt{U(U+2m-1)}}{1-U-m} \end{array}$$ (1)

RCP can also be expressed in a form more familiar in biology if dyad frequencies are considered as genotype frequencies for a gene with two alleles. RCP^2^ is 4*MU*/*H*^2^, which is expected to equal 1 under the Hardy-Weinberg equilibrium [21, 22]. Thus, RCP can be considered as a measure of deviation from random expectations.

**Fig 1:**
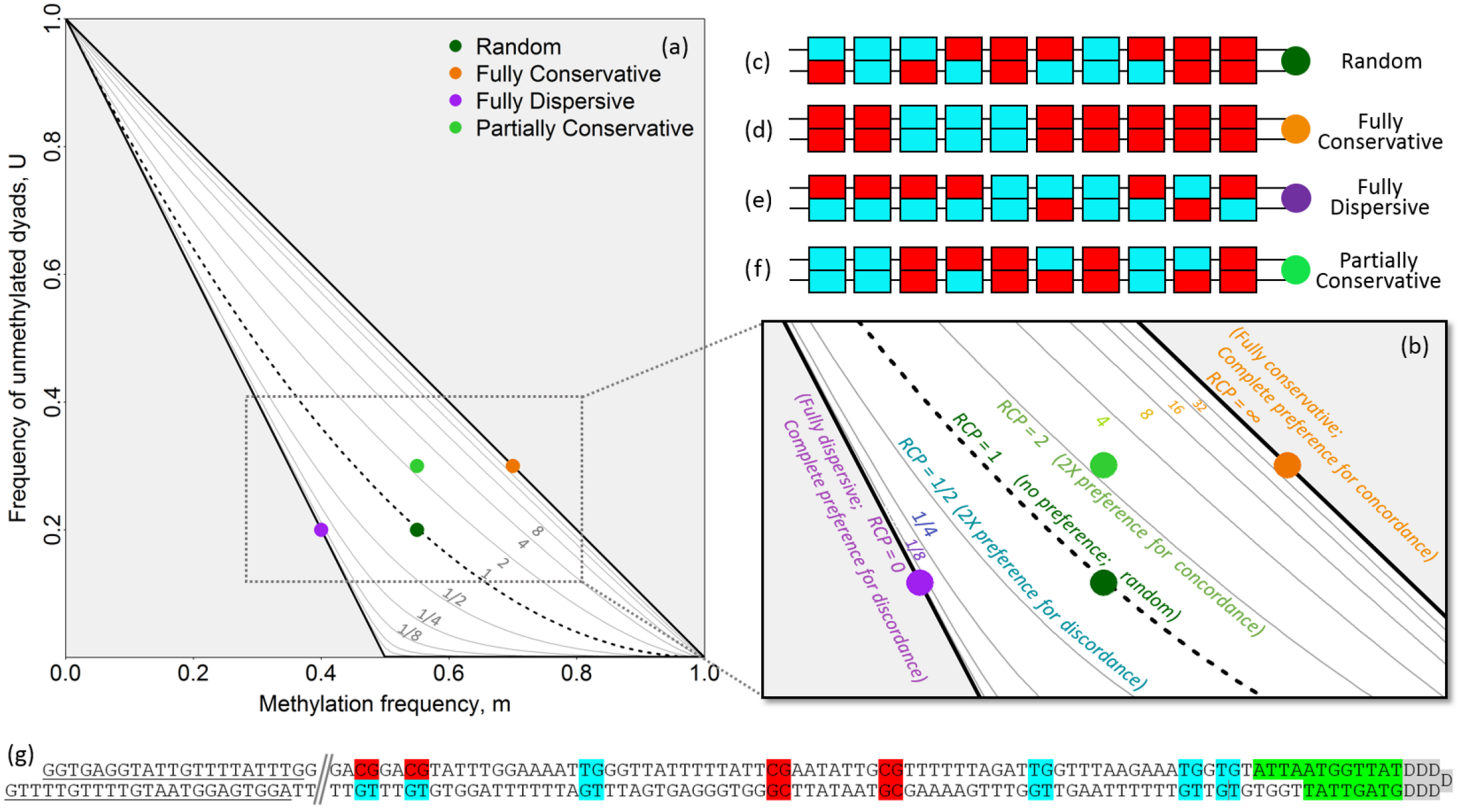
Characterizing methylation systems using double-stranded DNA methylation patterns. **(a)** Frequencies of methylated cytosines (*m*) and unmethylated dyads (*U*) locate each data set on the continuum from complete preference for concordance to complete preference for discordance. Ratio of Concordance Preference (RCP) is indicated for each contour line. **(b)** Expanded view. For this schematic, individual double-stranded methylation patterns **(c-f)** are used to illustrate different methylation configurations that lie along this continuum. Individual patterns, with methylated and unmethylated cytosines indicated in red and blue, respectively, can reflect **(c)** a random methylation system; **(d)** a fully conservative system, with complete preference for generating concordant dyads; **(e)** a fully dispersive system, with complete preference for generating discordant dyads (partial preference for dispersive placement is also possible); or **(f)** a partially conservative system, with more concordant dyads than expected under random processes, but fewer than expected under fully conservative processes. **(g)** A representative partial double-stranded DNA methylation pattern collected using hairpin-bisulfite PCR. The experiment-specific batchstamp is shown in green, and can be used to monitor for PCR contamination; the molecule-specific barcode shown in gray, generalized as “DDDDDDD” and a random sequence of non-cytosine nucleotides, can be used to identify redundant sequences. The batchstamp and barcode are encoded by the hairpin oligonucleotide used to join the top and bottom strands. Primer-binding sites are underlined at the left end of the molecule.

The random expectations, for which RCP = 1, are met both with truly random placement of methyl groups, and with equal contributions from processes operating with strong preference for concordance and processes operating with strong preference for discordance. Under either of these circumstances, the frequency of unmethylated dyads is given by *U* = (1 − *m*)^2^, leading to dyad frequencies as expected under the binomial distribution (Fig 1a and 1b dashed curve; Fig 1c). A system in which methyl groups are added *de novo* without regard to the methylation state of the other strand [23], such as one dominated by the activity of mammalian Dnmt3s, behaves largely in accordance with random expectations.

One set of deviations from the random expectation is characterized by preference for concordant placement of methyl groups, such that the two classes of concordant dyads—fully methylated and fully unmethylated—are more frequent than expected under the random model. This situation occurs under conservative systems of methylation where strong contributions from maintenance-like processes, such as the activity of Dnmt1 in mammals [11, 13, 24], lead to high frequencies of concordant dyads. In the extreme form of this deviation from random, methyl groups are observed only in fully methylated dyads (Fig 1d), such that unmethylated dyads occur at frequency *U* = 1 − *m* (upper diagonal line in Fig 1a and 1b).

The other set of possible deviations from random is characterized by preference for discordant placement of methyl groups, leading to an overabundance of hemimethylated dyads. This situation occurs under dispersive systems of methylation such as those that yield transient hemimethylation following DNA replication and prior to daughter-strand methylation, and perhaps in genomic regions undergoing demethylation during periods of epigenetic transition. When methylation is maximally dispersive and methylation frequency *m* is less than 0.5, all dyads with methylation will be hemimethylated (Fig 1e), such that *U* = 1 − 2*m* (lower diagonal line in Fig 1a and 1b); when *m* is greater than 0.5, not all methyl groups can be accommodated in hemimethylated dyads, and so a combination of hemimethylated and fully methylated dyads—but no unmethylated dyads—is expected (lower horizontal line in Fig 1a).

The two extreme deviations from random form the boundaries of the comprehensive space of possible configurations of methylation at symmetric motifs (Fig 1a). Sets of double-stranded methylation patterns fall on the continuum between the extreme expectations (Fig 1b), and can be located within this space to characterize the strategy of information transfer employed to give rise to a given data set, ranging from conservative to dispersive.

As noted above, a system with an RCP value of 1 has no preference for either concordance or discordance of methylation, and is analogous to the distribution of genotype frequencies at a two-allele locus in a population that is at Hardy-Weinberg equilibrium [21, 22]. An RCP value of 2 indicates two-fold preference for concordance, while an RCP of $\frac{1}{2}$ indicates two-fold preference for discordance. RCP approaches infinity for systems that have complete preference for concordant dyads. At the other extreme, RCP approaches 0 (i.e., $\frac{1}{\infty }$) for systems that have complete preference for discordant dyads.

For the examples analyzed here, data for different loci and cells range from complete concordance to near-random, along the RCP spectrum. Complete discordance is found as a transient condition of adenine methylation at the *ori* locus in *Escherichia coli*, and serves to regulate the timing of reinitiation of DNA synthesis [25, 26]. Adenine methylation in *E. coli* generally occurs at symmetric sites, such as the GATC motif within the *ori* locus, and can be assessed by PacBio sequencing [27]. Thus, a broad spectrum of concordance preference can exist in organisms, and can be quantified and evaluated by RCP.

For large and intermediate-size data sets, the resolution of RCP is high across the range of possible methylation frequencies, although the resolution declines as *m* approaches 0 or 1, such that RCP cannot be inferred for completely methylated or unmethylated genomic regions. Nonetheless, RCP can usually be inferred with high confidence using data from only a few hundred dyads. Our new approach therefore requires far fewer sequences to estimate concordance preference than do methods that focus on inferring rates for specific enzyme activities [24, 28].

We apply RCP to investigate further the conclusion of Shipony *et al.* [16] that methylation in cultured stem cells is dominated by non-conservative processes, with little or no preference for concordance. Using double-stranded methylation patterns collected by our group, by Arand *et al.* [14, 17], and by Zhao *et al.* [15], we assess and compare methylation concordance in cultured human and murine stem cells, as well as in murine cells undergoing early developmental transitions that give rise to totipotent embryonic cells.

### Differentiated cells strongly prefer concordant DNA methylation

**Fig 2:**
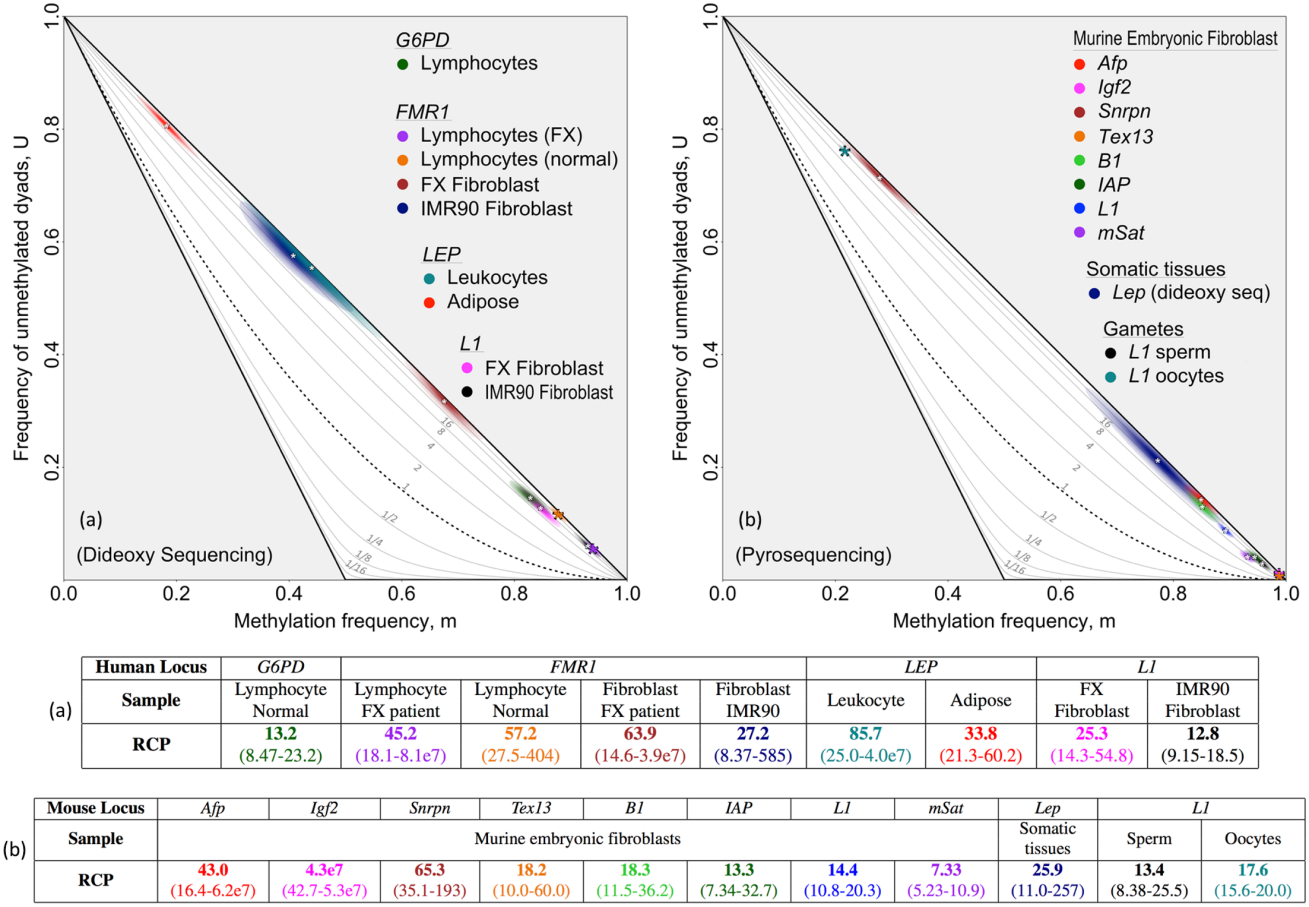
Inferring RCP for loci in differentiated human and murine cells. Methylation in human and murine differentiated cells was consistently inferred to have strong contributions from conservative processes, using data sets that span a wide range of methylation frequencies. For each locus or multi-copy family, we inferred the RCP point estimate of the *m*, *U* pair and the two-dimensional confidence region, determined by the uncertainty in the two variables (S7 Text). The intensity of coloration at a given point in a confidence region reflects the confidence level at that point. The *m*, *U* point estimates for most data sets are indicated with white asterisks, and the corresponding RCP values are given in the associated table. A larger, colored asterisk is used when the confidence interval of a data set is too small to be readily visible. RCP point estimates and bias-corrected bootstrap confidence intervals are shown in figure-associated tables. **(a)** Three single-copy human loci—*G6PD*, *FMR1*, and *LEP*—and one human repeat family, *L1*, all from various tissues as indicated. **(b)** Four single-copy loci and four repeat families—*Afp*, *Igf2*, *Snrpn*, *Tex13*, *B1*, *IAP*, *L1*, and *mSat*—from murine embryonic fibroblasts, one single-copy murine locus—*Lep*—from somatic tissue, and one repeat family—*L1*—from murine gametes. Data in (a) were collected using hairpin-bisulfite PCR and dideoxy sequencing, and taken from published [24, 28, 29, 41] and previously unpublished work (S3 Table). Data in (b) were collected by Arand *et al.* [14] using hairpin-bisulfite PCR and pyrosequencing, with the exception of murine *Lep* for which data from somatic tissues were collected by Stoger [29], using dideoxy sequencing. Compared to dideoxy sequencing, pyrosequencing can provide greater sequencing depth, but yields considerably shorter reads. Our analyses of these data sets applied bootstrapping approaches and accounted for inappropriate and failed conversion of methylcytosine using methods described in Supporting Information (S4 Text). Dyad counts, conversion-error rates, and inferences for methylation frequencies and RCPs are summarized in S3 and S4 Tables.

Our previous work with human single-copy loci in uncultured, differentiated cells revealed a substantial role for maintenance methylation, a conservative process, with a comparatively minor role for non-conservative *de novo* processes [24, 28]. We therefore anticipated that RCP analysis of double-stranded methylation patterns from such cells would indicate substantial preference for concordant methylation states. Data published previously for *G6PD*, *FMR1*, and *LEP*, in uncultured differentiated cells and new data presented here for *FMR1* in cultured, human differentiated cells represent blood, connective, and adipose tissues. We applied RCP analysis to these data sets and found 13.2- to 85.7-fold preferences for concordant methylation. This confirms, as anticipated, that methylation is predominantly conservative in these differentiated cells (Fig 2a; see table accompanying Fig 2 for approximate 95%CIs). We note that there is a good correspondence between RCP and hemi-preference ratio, a statistic we computed for the same data sets in the previous study [24] (further discussion in S2 Text).

We also found a substantial role for conservative methylation processes at single-copy loci in both cultured and uncultured murine differentiated cells. Data sets from Arand *et al.* [14] for *Afp*, *Igf2*, *Snrpn*, and *Tex13* from murine embryonic fibroblasts (MEFs), and from Stöger [29] for *Lep* from somatic tissues, gave RCP point estimates indicating a greater than 18-fold preference for concordant methylation (Fig 2b).

Do multi-copy sequence families also have high preference for concordant methylation in differentiated cells? We inferred RCP for four repeat families—*B1*, *IAP*, *L1*, and *mSat*—using murine data collected by Arand *et al.* [14]. Three of these families were found to have preference for concordant methylation in the same range inferred for single-copy loci (RCP point estimates between 14.4 and 18.3; Fig 2b). The fourth—*mSat*—had an RCP estimate of 7.33, lower than other families and single-copy loci examined in MEFs, but still indicative of strong preference for concordant methylation. For human cells, data from two independent lines of cultured embryonic fibroblasts were available for the repeat family *L1*. Inferred RCP values were within the range found for single-copy loci in both human and murine differentiated cells (Fig 2a).

Overall, we find appreciable preference for concordance across a diverse group of data sets from differentiated cells. These sets span a more than five-fold range in methylation frequency, underscoring the independence of RCP from *m*, and, more generally, highlighting the capacity of methylation systems to propagate specific epigenetic states even when methylation is sparse. We conclude that preference for concordant methylation, albeit to variable degrees, is present in differentiated cells across broad classes of genomic elements, cell and tissue types, and culture states.

### Concordance preference is reduced but still substantial in cultured stem relative to differentiated cells

We next ask whether substantial preference for concordance, as we infer above for differentiated cells, is also evident in data from cultured stem cells. In doing so, we compare our findings using RCP to the expectation from Shipony *et al.* [16] that methylation in such stem cells occurs primarily through non-conservative, random processes.

**Fig 3:**
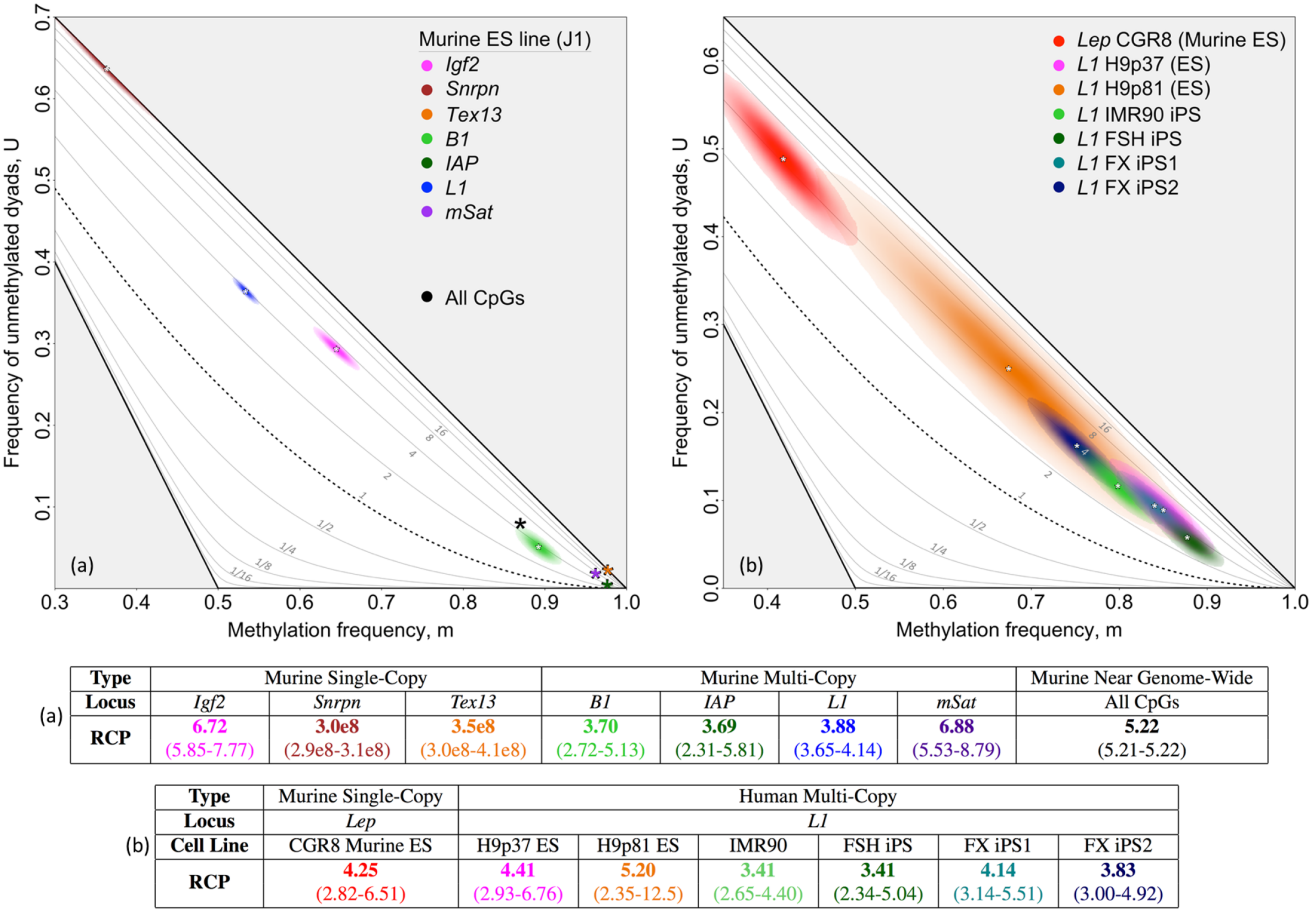
Inferring RCP in undifferentiated human and murine stem cells. Methylation patterns in undifferentiated, cultured human and murine stem cells were consistently inferred to have substantial contributions from conservative processes, with concordance greater than the random expectation. RCP point estimates and biased-corrected bootstrap confidence intervals are shown for individual loci and “All CpGs”. **(a)** Three single-copy loci—*Igf2*, *Snrpn*, and *Tex13*—as well as four multi-copy loci—*B1*, *IAP*, *L1*, and *mSat*—from murine ES J1 cells were assayed by Arand *et al.* [14]. “All CpGs” data, collected by Zhao *et al.* [15]), reflect methylation at 17.3% of CpG dyads in the murine genome (S5 Table). Data from both Arand *et al.* [14] and Zhao *et al.* [15] were collected using hairpin-bisulfite PCR and pyrosequencing. **(b)** Human *L1* and *LEP* data were collected using hairpin-bisulfite PCR and dideoxy sequencing (data from published [29] and previously unpublished work (S3 Table)). Our analyses of these data sets applied bootstrapping approaches and accounted for inappropriate and failed conversion of methylcytosine using methods described in Supporting Information (S4 Text). Dyad counts, conversion-error rates, and inferences for methylation frequencies are given in S3, S4 and S5 Tables.

The broadest data set available for our analysis comes from the near-genome-wide double-stranded methylation data presented by Zhao *et al.* [15]. These data give an inferred RCP of 5.22 for “all CpGs” in DNA from undifferentiated, cultured murine ES cells (Fig 3a; S5 Table). For other classes of genomic elements in these near-genome-wide data [15], we infer RCP values of 4.31 or greater (S5 Table). These RCP values are significantly higher than 1, the value predicted under Shipony *et al.*’s proposal of dynamic methylation (*p* < 10^−16^, maximum likelihood comparison tests (MLCTs); S8 Text).

We next ask whether our inference of appreciable concordance preference in the murine ES cell line used by Zhao *et al.* [15] reflects a general property of cultured lines of undifferentiated stem cells, both murine and human. For the murine ES line, J1, double-stranded methylation data collected by Arand *et al.* [14] were available for four single-copy loci and four repeat families. Seven of the eight genomic regions—*Igf2*, *Snrpn*, *Tex13*, *B1*, *IAP*, *L1*, and *mSat*—had RCP values greater than 3.69 (with minimum 95%-CI lower bound of 2.31), still indicative of substantial preference for concordant methylation (Fig 3a). One single-copy locus, *Afp*, had a methylation level too high, 0.99, to permit reliable inference of RCP. Murine double-stranded methylation patterns for the four repeat families were available for two more stem cell lines, E14 and WT26 [14]. These additional repeat-family data sets, too, had RCP values significantly greater than 1, although one data set, *mSat* in WT26, had an RCP value closer to 1 than did others (*p* = 0.045, one tailed bootstrap test (BT); S7 Text). Data were available for a single-copy locus, *Lep*, for a fourth murine ES line, CGR8. Here, too, RCP was significantly greater than 1 (*p* < 10^−16^, one-tailed BT).

Human stem cell lines also exhibited aprpeciable preference for concordant methylation. All six of the human stem and iPS cell lines that we examined, when grown under non-differentiating conditions, gave RCP point estimates for the repeat family *L1* that are between 3.41 and 5.20. For all of these cell lines, outer bounds of the approximate 95% confidence intervals fall between 2.34 and 12.5 (Fig 3b; S3 Table). Together, these values reveal concordance preference that is reduced relative to differentiated cells, but still greatly exceeds expectations under random placement of methyl groups (*p* < 10^−16^, one-tailed BT).

We now consider the possibility that spontaneous differentiation had produced subpopulations of cultured stem cells that might account for the inference of RCP values substantially greater than 1 at the seven different loci and genomic elements examined. Our calculations revealed that a possible subpopulation of differentiated cells operating at much higher RCP than that of undifferentiated cells would need to comprise more than 50% of the population to account for our finding (S9 Text). Morphological inspection of the cultured human stem cells under non-differentiating conditions did not suggest the presence of a substantial subpopulation of differentiated cells in any of these lines.

We conclude that RCP values significantly greater than 1 are a consistent feature of cultured embryonic stem cells, and exist across a broad set of stem cell lines, genomic locations and element categories.

### Preference for concordance is minimal or absent in ES cells deficient in maintenance methylation activity

Our finding of substantial preference for methylation concordance in data from cultured, undifferentiated stem cells contrasts with the inference of Shipony *et al.* [16] that DNA methylation in such cells is dominated by non-conservative, random processes. This disparity led us to ask whether our approach here for data acquisition and analysis is indeed capable of identifying sets of methylation patterns established under exclusively random processes, which are expected to yield RCP values of 1 (see “Ratio of Concordance Preference is Defined …”, above).

To assess this capacity, we consider methylation patterns from two murine embryonic stem cell lines that have impaired maintenance methylation: a *Dnmt1* knockout (KO) line and an *Np95* KO line. The Dnmt1 enzyme is principally responsible for addition of methyl groups to daughter-strand CpGs complementary to CpGs methylated on the parent strand [11, 24, 30]; Np95 facilitates interaction of Dnmt1 with these hemimethylated sites [31]. Absence of either protein is therefore predicted to markedly diminish maintenance activity. If our approach is able to detect essentially random placement of methyl groups, RCP values in these knockout lines should be ∼1 for loci for which Dnmt1, aided by Np95, is principally responsible for conservative methylation.

**Fig 4:**
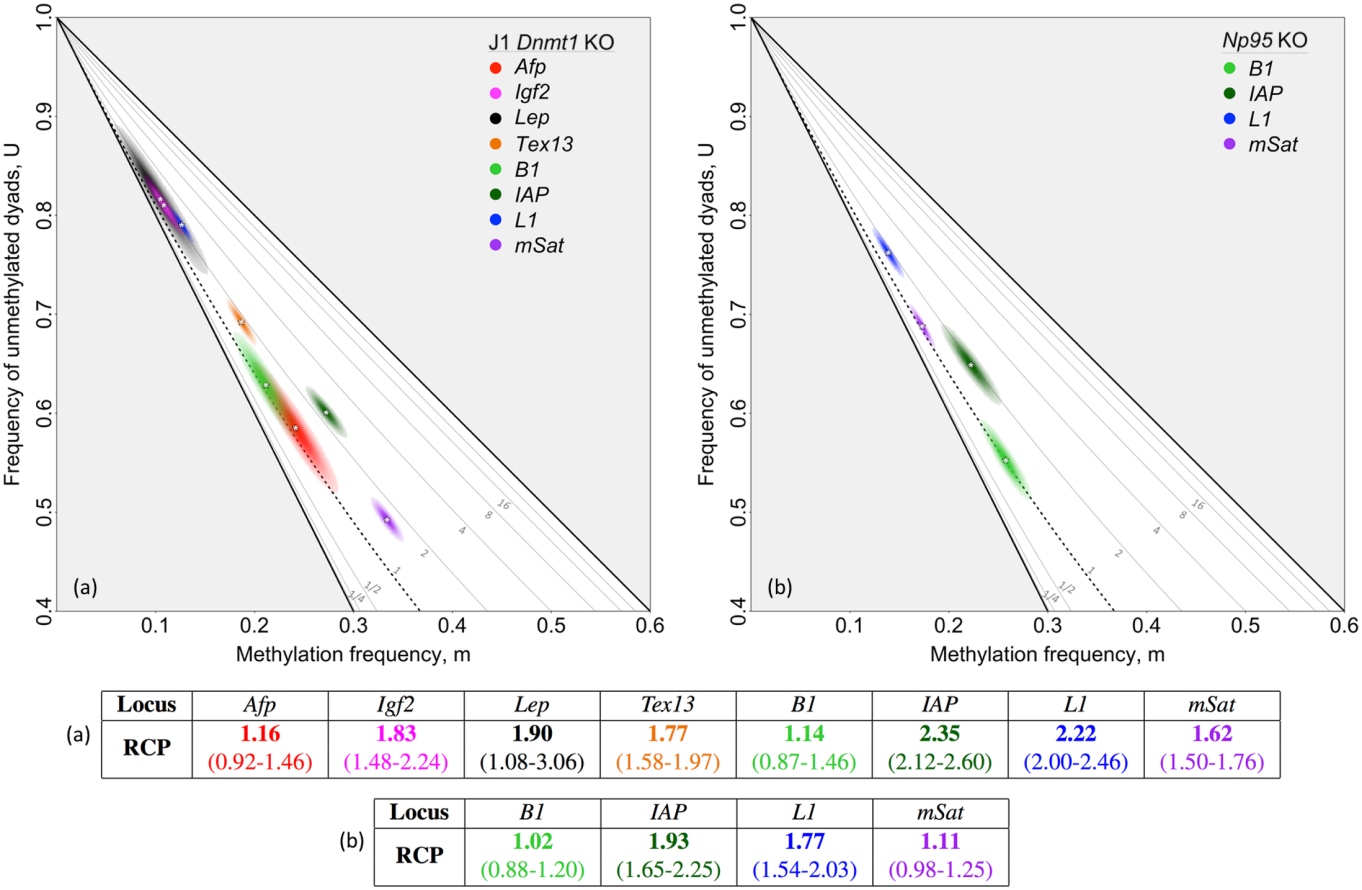
Inferring RCP in *Dnmt1* knockout and *Np95* knockout ES cells. (**a**) In the absence of the Dnmt1 maintenance methyltransferase, some loci in cultured murine ES cells had RCP values close to 1, the random expectation. Data from *Lep*, collected using hairpin-bisulfite PCR and dideoxy sequencing, are from Al-Alzzawi [32]. Data from seven additional loci are from Arand *et al.* [14], who used hairpin-bisulfite PCR and pyrosequencing. Our analysis of these published data revealed two of the eight loci analyzed—*Afp* and *B1*—to have very low RCP values not significantly different from 1. (**b**) In the absence of Np95, a protein critical for recruiting Dnmt1 to hemimethylated regions in newly replicated DNA, one of the four loci analyzed—*B1*—was inferred to have a very low RCP value, not significantly different from 1. Our analyses of these data sets from Arand *et al.* [32] applied bootstrapping approaches and accounted for inappropriate and failed conversion of methylcytosines using methods described in S4 Text. Point estimates and approximate 95% confidence intervals on RCP are given above, and also along with conversion-error-rate estimates in S3 and S4 Tables.

Significant reductions in RCP were inferred for all single-copy loci and repeat families examined in *Dnmt1* and *Np95* KO lines [14, 32], compared to the parent stem-cell lines. Some reductions were sufficient to bring RCP values in the knockout lines to that expected for random placement of methyl groups: one single-copy locus—*Afp*—in the *Dnmt1* KO line and one repeat family—*B1*—in both knockout lines had RCP values not significantly different from 1 (*Afp* in *Dnmt1* KO: 1.16, *p* = 0.10; *B1* in *Dnmt1* KO: 1.14, *p* = 0.17; *B1* in *Np95* KO: 1.02, *p* = 0.38; one-tailed BTs; Fig 4). These findings in the two mutant cell lines reveal that RCP analysis is, indeed, able to detect methylation established with random placement of methyl groups, and thus with little or no preference for concordance or discordance.

The ability of RCP to detect random methylation has important implications for our work. First, we can conclude that our inference of persistent preference for concordant methylation in cultured stem cells reflects a *bona fide* property of those cells, rather than an artifact of our approach. Second, we can infer from our finding of RCP >1 for nine of the twelve data sets examined in the *Dnmt1* and *Np95* KO lines that methyltransferases other than Dnmt1 can contribute to conservative methylation. This inference is consistent with earlier conclusions that contributions of Dnmt3s can include low levels of maintenance activity [19, 24, 33]. Nonetheless, knocking out one or both of Dnmt3s generally increased the relative contributions of conservative processes (S2 Fig; S10 Text), highlighting the *de novo* properties of the Dnmt3s.

### Concordance preference increases upon differentiation of ES cells, and decreases upon dedifferentiation

**Fig 5:**
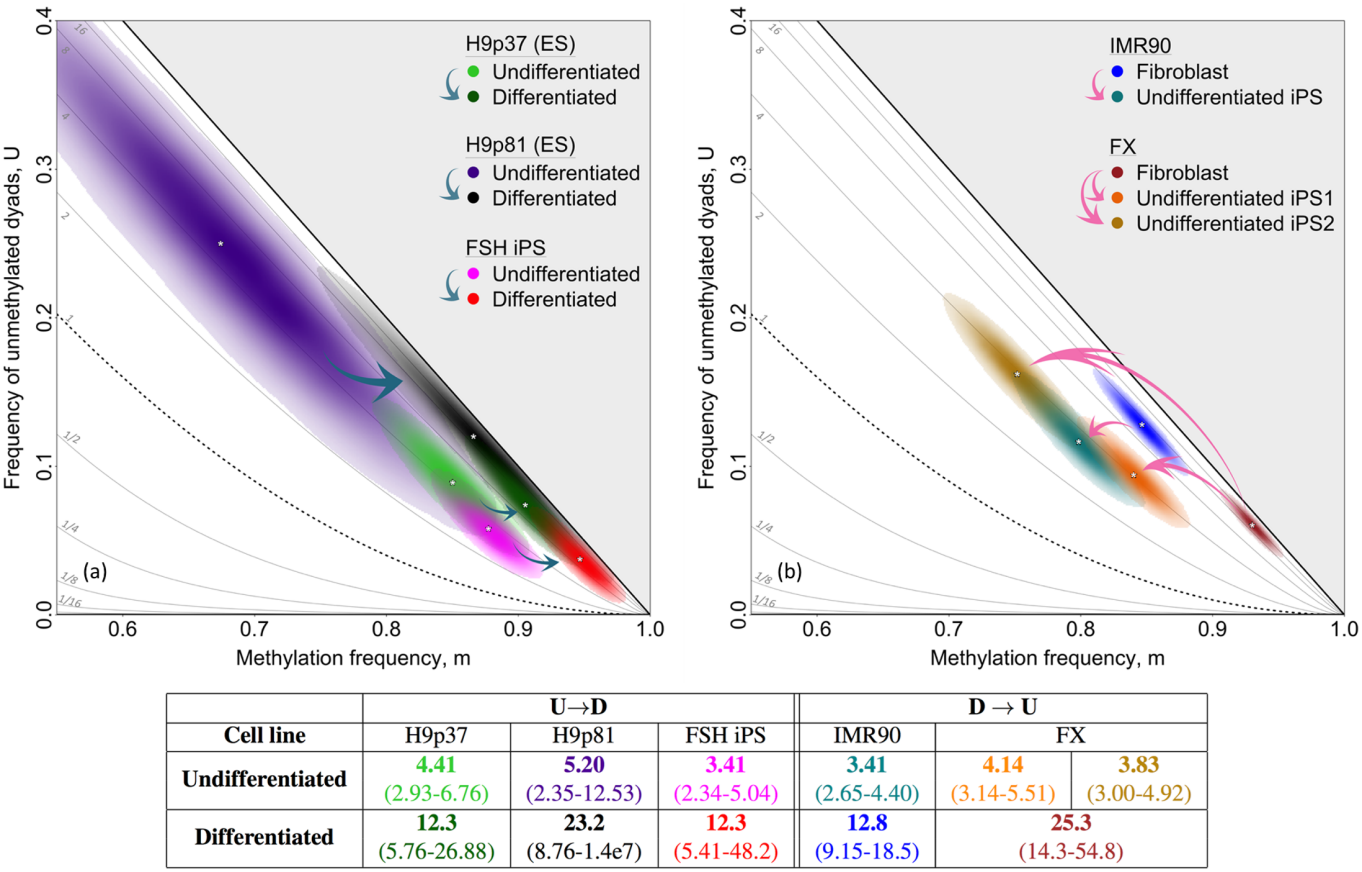
Shifts in RCP of *L1* elements upon differentiation of cultured human ES cells and dedifferentiation of cultured fibroblasts. RCP values of *L1* elements in cultured stem cells grown under non-differentiating conditions are compared to those for the same cells grown under differentiating conditions. RCP values for progenitor fibroblast lines are compared with those for their descendent iPS cells. Blue arrows indicate differentiation and pink arrows indicate dedifferentiation. **(a)** Undifferentiated cells from each of three human ES and iPS lines had only moderate preference for concordance. Upon differentiation, their RCP values shifted in parallel toward stronger preference for concordance. **(b)** Differentiated human fibroblast lines had substantial preference for concordance. Upon dedifferentiation to iPS cells, RCP values were reduced, indicating diminished preference for concordance. We present data for two different iPS lines established independently from the fibroblast line, FX. These iPS lines differed in their methylation frequency, *m*, at *L1* elements, but had similar RCP values. Some data were previously shown in Figs 2 and 3, and are included again here to illustrate more directly the relationships of *L1* methylation in the differentiated and undifferentiated cultured cells. We applied bootstrapping approaches and accounted for inappropriate and failed conversion of methylcytosine using methods described in S4 Text. Point estimates of RCP, with approximate 95% confidence intervals, are shown above, and also with dyad counts, conversion-error rates, and inferences for methylation frequencies in S3 and S4 Tables.

Our initial examination of RCP values in differentiated cells as compared to cultured stem cells suggests that RCP is altered through the differentiation process (Figs 2 and 3). Would significant RCP increases be observed for individual cell lines transitioning between differentiation states? We first asked whether RCP values change when undifferentiated human ES and iPS cells are grown under differentiating conditions (see Materials and methods). We inferred RCP at the promoter of *L1* elements of cultured human iPS and ES cells, inferring values for two different passages of the latter cell line. Upon differentiation, RCP values for all three of these cell lines increased significantly (*p* = 0.004, H9p37; *p* = 0.025, H9p81; *p* = 0.0005, FSH iPS; two-tailed permutation tests (PTs); S7 Text), and approached the lower boundary of the confidence region inferred for single-copy loci in differentiated somatic cells (Figs 2 and 5a). Using near-genome-wide data for cultured murine cells [15], we inferred significant RCP increases upon cell differentiation for most genomic elements (*p* < 10^−16^, MLCTs), with the exception of low-complexity and satellite DNAs (S5 Table). These RCP increases were greatest at promoters, CG islands, and CG shores, and were more modest at other regions. We conclude that the onset of differentiation in cultured human and murine cells is associated with a shift towards a greater role for conservative processes.

Does the dedifferentiation that occurs in culture upon production of an iPS line from a differentiated cell have an opposite effect on concordance preference? To address this question, we compare methylation at *L1* elements in three iPS lines to that in the two cultured human fibroblast lines from which they were derived. As predicted, RCP values for all three iPS lines were much reduced compared with values observed for the parent fibroblast lines (*p* < 10^−16^, two-tailed PTs; Fig 5b). Dedifferentiation in tissue culture is thus associated with a shift in DNA methylation toward a greater role for non-conservative processes. It will be useful to investigate whether changes in methylation systems as measured by RCP drive or merely reflect the cellular differentiation process.

### Murine embryos mirror the transitions in concordance preference observed in cultured cells

The ∼3-fold-or-greater preference for concordant methylation we infer in many different cultured ES and iPS cell lines (Fig 3a; S3 Table) far exceeds the concordance expected under the null hypothesis that methyl groups are placed at random. Here we ask whether this appreciable preference for concordance is an artifact of growing stem cells in culture, or whether it is shared by uncultured stem cells taken directly from an embryo.

**Fig 6:**
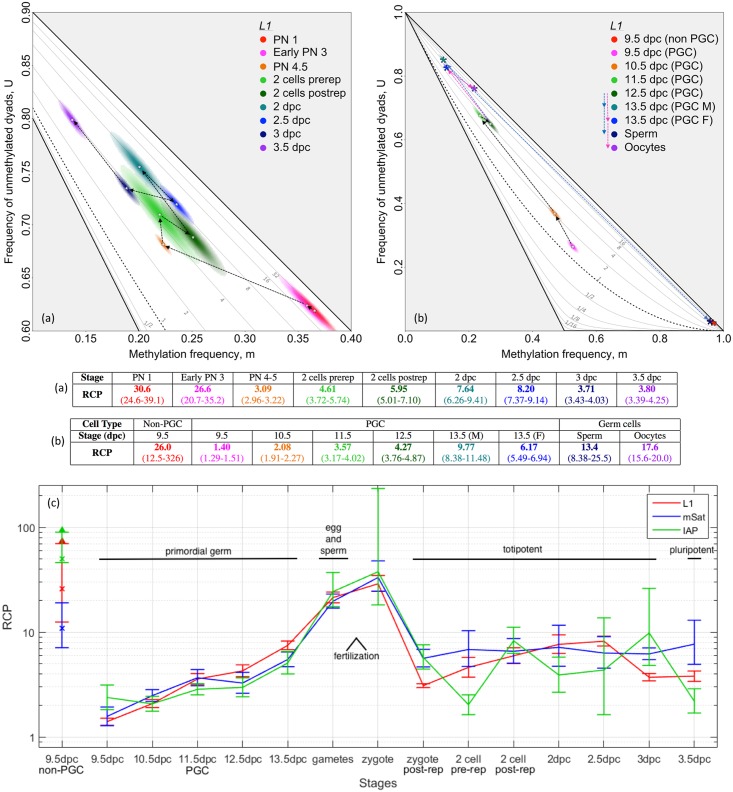
Shifts in RCP during murine embryonic and germ-cell development. Major transitions in RCP values occur during early embryonic and primordial germ cell (PGC) development. RCP point estimates and approximate 95% bias-corrected bootstrap confidence intervals are given in the associated tables and in (c). **(a)** Transitions from early pronuclear stages (1 and 3) to late stages (4-5) were accompanied by a sharp decrease in RCP at *L1* elements, to a level similar to that observed in cultured stem cells (Fig 3). Further embryonic development was accompanied by minor increases and subsequent decreases in methylation and RCP values. **(b)** In PGCs at the earliest stage for which data are available, 9.5 days post conception (dpc), *L1* elements had RCP values that were unusually low but still significantly greater than 1 (*p* < 10^−16^). RCP values increased during PGC maturation to stage 13.5 dpc even as methylation frequencies decreased by more than 50%. RCP values for eggs and sperm, shown previously in Fig 2b), are included here to highlight the transition from early primordial germ cells to terminally differentiated gametes. **(c)** Tracking RCP at repeat families during development. We inferred RCP for the embryonic and differentiated stages for which data were published by Arand *et al.* [17]. The topology of our longitudinal RCP plot highlights the transitions also evident in Arand *et al.*’s plot of the percentage of hemimethylated CpG dyads relative to all methylated CpG dyads (Figure 6b in [17]). Their metric captures relative shifts in concordance, but, in contrast to RCP, does not include a null model enabling quantitative comparison of inferred concordance across data sets with disparate methylation frequencies [17]. Point estimates and approximate 95% confidence intervals for all but one data set were estimated by bootstrapping and applying the BCa correction (S7 Text). Dyad counts, methylation frequencies, and conversion error rates are in S3 Table. In cases where data were available for multiple replicates, dyad frequencies were pooled (S6 Text). For one data set, 3 dpc at *mSat*, double-stranded sequences and methylation patterns were not available to us for bootstrap analysis; we therefore used a likelihood-based method for the estimation of confidence intervals (S8 Text).

We first consider whether totipotent cells from an embryo have evidence of conservative processes. We applied RCP to double-stranded methylation patterns collected by Arand *et al.* [17] for three multi-copy loci in mouse embryos: *L1*, *mSat*, and *IAP*. Our analyses revealed that these totipotent embryonic cells, from post-replicative zygote to morula stage (through 3 days post conception, dpc), also exhibit moderate preference for concordance. Each of the eighteen data sets we considered yielded an RCP point estimate greater than 1, and a confidence interval that excludes 1 (*p* < 0.005; Fig 6a; S4 Table).

Pluripotent stem cells from mouse embryos (gastrula, 3.5 dpc) also exhibit moderate preference for concordant methylation for all three of the multi-copy loci examined (*p* < 10^−16^; Fig 6a; S4 Table). Thus, we conclude that moderate preference for concordance is an epigenetic feature of uncultured embryonic stem cells of disparate developmental potential, and is not an artifact of the establishment of embryonic stem cells in culture.

Though RCP values for stem cells from embryos clearly indicate some preference for concordance, the extent of this preference is lower than for differentiated cells at most of the loci examined (Figs 2 and 5). Does this lower preference for concordance originate in gametes, or does it instead arise post-fertilization? To address this question, we expanded our inference of RCP values for sperm and oocytes from exclusively *L1* (Fig 2b) to all three loci analyzed by Arand *et al.* [17]. At the three multi-copy loci, RCP values for gametes are within the range observed for other differentiated cells, with average point estimates ranging from 13.4 to 45.0 (Fig 6b; S4 Table). This high preference for concordance in gametes implies that the lower RCP values characteristic of zygotes and stem cells must arise post-fertilization rather than in gametes.

To pinpoint the timing of this transition to lower RCP values observed in stem cells, we consider data from post-fertilization nuclei and cells [17]. Data available for pronuclear stages 1, 2, and 3 revealed high RCP values, similar to those observed in gametes. In pronuclear stages 4-5, however, there was an abrupt transition to lower RCP values in the range observed for totipotent stem cells (Fig 6; S4 Table).

Is this transition dependent on the DNA replication event that occurs from pronuclear stage 3 to stages 4-5? To address this question, we assess data from Arand *et al.* [17], in which aphidicolin was used to block DNA replication in the fertilized egg. Our RCP analysis reveals that methylation patterns at *L1* and *mSat* in these treated cells, while having somewhat lower RCP values relative to pronuclei at earlier stages, had not undergone the major reduction in RCP that we infer for unmanipulated pronuclei at stages 4-5 (*p* < 10^−16^, two-tailed PTs; S4 Table). Thus, the shift to lower RCP in stem cells following fertilization appears to require either DNA replication or some later event that is itself replication-dependent. This conclusion is consistent with the inference of Arand *et al.*, using a different metric [17], that DNA replication in the zygote plays a pivotal role in methylation dynamics.

This change from high RCP values in gametes and early pronuclei to lower values in post-replicative zygotes and descendant stem cells was markedly abrupt. Are other major transitions in RCP similarly abrupt, or do some occur gradually, perhaps over many cell divisions? Murine primordial germ cells (PGCs), in their maturation to differentiated gametes, offer an opportunity to approach this question [34, 35]. Using data from Arand *et al.* [17], we infer that RCP values for PGCs increased by factors ranging from 2.5 to 5 during their progression from 9.5 dpc to 13.5 dpc. This increase was not sudden, but occurred over the four-day period, and so spanned an interval of substantial cell proliferation [34] (Fig 6b). The murine embryo data thus provide examples of both abrupt and gradual transitions in RCP through development (Fig 6c).

The RCP values for PGCs at 9.5 dpc, the earliest stage for which data were reported by Arand *et al.* [17], were strikingly low: 1.40 for *L1*, 1.57 for *mSat*, and 2.38 for *IAP*. Nonetheless, confidence intervals for all three loci indicated RCP values greater than 1 (*p* < 2 × 10^−5^), the value expected under wholly random placement of methyl groups, indicating persistent, low-level preference for concordance. This low, residual preference for concordance in maturing PGCs perhaps reflects both the epigenetic memory needed to maintain the poised state of stem cells, and the epigenetic flexibility required for the production of differentiated gametes.

The first few rounds of PGC division involve developmental reprogramming and commitment [35], and establishment of lineage-specific gene expression patterns. The 3.5-fold average increase of RCP across these divisions (Fig 6; S4 Table) mirrors the 3.6-fold average increase in RCP values that occur when cultured ES cells are subjected to differentiating conditions (Fig 5; S3 Table).

There is, however, a critical difference between the trajectories for proliferating PGCs and differentiating ES and iPS cells in culture. When cultured cells were differentiated, their methylation frequencies increased. By contrast, methylation frequencies for PGCs declined across early rounds of division [36]. Seisenberger *et al.* [7], Arand *et al.* [17], and von Meyenn *et al.* [18] concluded that this reduction in methylation frequency is driven by partial impairment of maintenance methylation.

Our RCP framework permits a closer look at the likely extent of this proposed maintenance impairment. If maintenance methylation were completely absent and no other methylation processes were active, passive, fully dispersive demethylation would occur. This would halve methylation frequencies and leave methyl groups only in hemimethylated dyads, yielding an RCP value of 0. By contrast, data from PGCs yielded RCP values significantly greater than 0, and even 1. Indeed, only cells treated with S-Adenosylmethionine-ase (RCP point estimate: 0.20, approximate 95% confidence interval: 0.15—0.26; S4 Table) yielded values close to 0. This is not surprising, as S-Adenosylmethionine-ase either impairs or eliminates a cell’s ability to methylate DNA, and so reveals RCP trajectories that would be observed with complete or nearly complete suspension of all methylation processes. Together these findings confirm that, while the RCP framework can detect very low RCP values, maturing PGCs retain conservative methylation processes, and that these processes occur at levels sufficient to outweigh any dispersive effects of passive demethylation.

### Concluding remarks

Because RCP makes no explicit enzymatic or mechanistic assumptions about the methylation machinery, it permits quantification and comparison of strategies for symmetric methylation across cell types, developmental periods, and organisms, despite variation in exact mechanisms. Application of RCP to double-stranded DNA methylation patterns reveals that preference for concordance in DNA methylation is a persistent though quantitatively variable feature of mammalian cells of disparate developmental potential. Specifically, we find that: (i) in cultured human and murine ES and iPS cells, preference for concordance is lower than in differentiated cells, but not absent; (ii) for cultured human stem cells, cellular differentiation is characterized by increasing preference for concordance, whereas, for cultured differentiated cells, dedifferentiation is characterized by declining preference for concordance; and (iii) during early murine development, transitions in RCP mirror those found in cultured cells, with pluripotent and totipotent stem cells showing appreciable concordance preference throughout. We also observe that substantial changes in RCP can be either abrupt, requiring only one DNA replication event, or gradual, occurring over multiple rounds of replication.

Although preference for concordance is substantial throughout early murine development, there is an instance of concordance preference near the expectation under entirely random processes. We infer RCP values close to, albeit significantly different from, 1 in the early primordial germ cell stage at the three repetitive element families examined by Arand *et al.* [17] (Fig 6). The instance of low, yet present, concordance preference may reflect both the epigenetic stability required to maintain the poised state of the stem cells and the epigenetic flexibility needed *en route* to production of functional gametes. Flexibility, indicated by RCP values near 1, may result from near-random processes or instead from a balance of conservative and dispersive methylation. Existing data and conclusions of Seisenberger *et al.* [7], Arand *et al.* [17] and von Meyenn *et al.* [18] are more consistent with the latter interpretation.

Our finding of moderate contributions from conservative DNA methylation processes in human and murine stem cells is seemingly contrary to the conclusion of Shipony *et al.* [16] that “dynamic” processes are dominant in cultured stem cells, even in regions where dense methylation is maintained. This apparent disparity may have arisen from differences between the temporal scales assayed by Shipony *et al.*’s approach and our own. The method of single-cell isolation and clonal expansion used by Shipony *et al.* estimates epigenetic memory from single-stranded data collected after 15 to 21 rounds of cell division. In contrast, our approach utilizes double-stranded DNA data to examine epigenetic memory over a single round of DNA replication. Evidence of preference for concordance, apparent in our comparison of DNA strands separated by one replication event, will be muted in comparisons of more distantly related molecules.

Short-term epigenetic memory, perhaps important for guiding cell-fate trajectories at early developmental stages, is at least partially achieved through preference for concordant DNA methylation. By contrast, over larger numbers of cell divisions, as sampled by Shipony *et al.* [16], such as for stem cells dividing in culture, preference for concordant methylation may be less important than other mechanisms of epigenetic memory. For example, regulation of promoter activity by DNA methylation can occur via an ensemble effect rather than by methylation of specific CG dyads within a promoter [37]. In such cases, propagation of exact methylation patterns may be less important than the density of methylation that influences gain or loss of methylation and states of transcriptional activity over many cell divisions [38]. Epigenetic mechanisms other than DNA methylation also contribute to epigenetic memory at various timescales. RCP analysis in combination with histone-modification data from ENCODE [39] and Roadmap [40] will provide unprecedented opportunities to infer interactions between DNA-methylation machinery and histone modification, the developmental timing of epigenetic stability, and its variation across the genome.

The value of RCP analysis will be enhanced and broadened by emerging DNA sequencing technologies that yield longer, more informative double-stranded methylation patterns. Longer sequence reads will enable inference of RCP for single cells, permitting study of cell-cell epimosaicism, such as arises in cancer [2] and other syndromes characterized by epigenetic heterogeneity and change [41, 42]. Some of these methods also reduce data corruption arising through errors in bisulfite conversion and amplification, and can distinguish between methyl- and hydroxy-methyl cytosine [43, 44]. High-resolution RCP estimates available through these advances will provide new insight into the flexibility and potential sensitivity of individual loci and cell types to environmental conditions encountered during embryogenesis and beyond.

## Materials and methods

### Ethics statement

Many of the sets of human DNA methylation patterns analyzed here were presented in previous publications, which include information on University of Washington Human Subjects approval for collection and use. These data include *G6PD* and *FMR1* from leukocytes of normal individuals [24, 28]; *FMR1* from males with fragile X syndrome [41]; *LEP* from male leukocytes and from female lymphocytes and adipose tissue [24, 29].

Human methylation patterns presented here for the first time were collected from: (i) FX iPS cell lines 1 and 2, which were developed at the University of Washington ISCRM facility from fibroblasts (line GM07730, Coriell Cell Repositories, Camden, NJ) of a male with a fragile X “full mutation”, using published methods [45]; (ii) iPS cell line IMR90, which was developed at the University of Washington ISCRM facility from the IMR90 somatic line established from fibroblasts (obtained from ATCC) of a normal female, using published methods [45]; (iii) FSH iPS cell line, which was developed at the University of Washington ISCRM facility from fibroblasts of an individual with Facioscapulohumeral dystrophy (FSHD), as previously described in [46]; and (iv) H9 human ES cells from NIH Embryonic Stem Cell Registry (WA09, H9 number 0062).

### Mathematical foundations of the RCP framework

Overview of the mathematical foundation is given in Results and Discussion, and developed further in S1 Text.

### Human cells and culture conditions

The six human ES and iPS cell lines for which we collected methylation patterns were derived from either embryos or fibroblasts described as normal [47] or from fibroblasts of individuals with disorders not known to affect the basic biochemistry of DNA methylation.

Cells were cultured in Dulbecco’s modified Eagle’s medium/Ham’s F-12 medium containing GlutaMax supplemented with 20 percent serum replacer (SR), 1 mM sodium pyruvate, 0.1 mM nonessential amino acids, 50 U/ml penicillin, 50 *μ*g/ml streptomycin and 10 ng/ml basic fibroblast growth factor (all from Invitrogen), and 0.1 mM *β*-mercaptoethanol (Sigma-Aldrich). hESCs were grown on *γ*-irradiated primary mouse embryonic fibroblasts (MEFs) and passaged using dispase (1.2 U/ml; Invitrogen). They were passaged onto Matrigel (Corning) without feeders in mTeSR1 (Stem Cell Technologies) for the final passages prior to analysis. Cells were differentiated by passage onto Matrigel in Dulbecco’s modified Eagle’s Medium supplemented with 20 percent fetal bovine serum and pen/strep. Images of our cultured stem cells grown under differentiating conditions confirmed their pluripotency.

### Murine cells and culture conditions

Methylation patterns from murine ES cells, and the origin and culturing of these cells, have previously been described [14, 15, 17, 32].

### Collection of double-stranded DNA methylation patterns using hairpin-bisulfite PCR

The DNA methylation patterns collected in our lab and analyzed here, both those published previously and those presented here for the first time, were collected using the hairpin-bisulfite PCR approach [13], with barcodes and batchstamps to authenticate each sequence [48]. Details for collection of each data set are given in S2 Table.

The data presented by Arand *et al.* [14, 17], and Zhao *et al.*, [15] and analyzed here, were collected in the absence of molecular batch-stamps and barcodes, raising the possibility that the reliability of those data sets is undermined by PCR clonality. However, both groups used alternate strategies that revealed that PCR clonality was not rampant. Zhao *et al.* found that essential features of data sets did not differ appreciably between the “real” data set collected conventionally, using PCR, and a test, PCR-free data set that excluded opportunities for clonality by including only one read from each locus, providing no evidence of impacts from PCR clonality (Hehuang Xie, personal communication). In turn, Arand *et al.* used molecular codes for several of their data sets, and, for the few data sets collected in the absence of such codes, observed appreciable heterogeneity among patterns, also hinting that data were not appreciably impacted by clonality (Julia Arand, personal communication).

## Supporting information

S1 Fig Markov chain used for the derivation of RCP. Derivation described in S1 Text.
(TIF)

S2 Fig RCP in *Dnmt3* knockout cells. **(a)** RCP values were inferred at the *Lep* locus for wildtype, *Dnmt3a* KO, and *Dnmt3b* KO murine ES cell lines, using data from Al-Azzawi [32]. **(b-i)** RCP values were inferred for eight additional loci for wildtype, *Dnmt3a* KO, *Dnmt3b* KO, and *Dnmt3a/b* double-KO murine ES cell lines, using data from Arand *et al.* [14].
(TIF)

S1 Table Comparison of RCP values inferred here to the DNMT1 hemi-preference ratios (HPR) inferred by Fu *et al.* [24]. Point estimates and approximate 95% confidence intervals are shown for both statistics. Fu *et al.* provides only lower bounds of approximate 95% confidence intervals.
(XLSX)

S2 Table Hairpin-linkage and PCR conditions for collection of double-stranded DNA methylation patterns. Entries shown are for patterns published here for the first time. R.E. refers to the restriction enzyme used to create the genomic overhang prior to ligation with a hairpin linker.
(PDF)

S3 Table RCP values and associated approximate confidence intervals inferred for the 24 data sets from our labs. We collected double-stranded DNA methylation patterns from two species—mouse and human—and several loci using bisulfite conversion under either low-molarity/temperature (“LowMT”) or high-molarity/temperature (“HighMT”) conditions [49]. For each data set, we counted methylated (*M*), hemimethylated (*H*), and unmethylated (*U*) dyads, and used these values to infer methylation frequency, *m*, unmethylated dyad frequency, *U*, and the ratio of concordance preference, RCP. Inferences for (*m*, *U*) and for RCP incorporated correction for conversion error, as described in S3 Text. We estimated approximate 95% confidence intervals by bootstrapping and applied the BCa correction to obtain bias-corrected confidence intervals and point estimates, as described in S7 Text. Both uncorrected and BCa-corrected intervals and point estimates are listed here.
(XLSX)

S4 Table RCP values and associated approximate confidence intervals inferred for data reported in Arand *et al.* [14, 17]. Dr. Julia Arand kindly shared raw double-stranded sequences for samples described in these publications. We accounted for failed and inappropriate conversion, as described in S3 Text, in our point estimates of *m*, *U*, and RCP. The inappropriate conversion rate of 0.031 was inferred because the bisulfite conversion conditions used by Arand et al. resembled lowMT conditions [49]. We estimated approximate 95% confidence intervals by bootstrapping and applied the BCa correction to obtain bias-corrected confidence intervals and point estimates, as described in S7 Text. Both uncorrected and BCa-corrected intervals and point estimates are listed here. For one sample, Arand *et al.* (2015) *mSat* 3dpc, only dyad counts, but no raw sequences, were available. We used the dyad counts to estimate the point estimate and the confidence interval for this sample while assuming independent sampling of dyads (S8 Text).
(XLSX)

S5 Table RCP values and associated approximate confidence intervals inferred for data reported in Zhao *et al.* (2014) [15]. Dr. Hehuang Xie kindly shared raw data on *M*, *H*, and *U* values for samples described in Figure S6 of Zhao *et al.*, and also provided information on error rates from bisulfite conversion: for the “Day 0” sample (cultured stem cells grown under non-differentiating conditions), the failed conversion rate was 0.011, and the inappropriate conversion rate was 0.0109; for the “Day 6” sample (cultured cells grown for 6 days under differentiating conditions), the corresponding error rates were 0.012 and 0.0099. Dr. Xie also commented, “‘All’ refers to the total CG dyads and ‘DNA’ refers to the CG dyads within ‘DNA repeat elements (DNA)’ annotated in the UCSC genome database.” For these data sets, whose individual sequence reads only contain 2 to 3 dyads on average, we assumed independent sampling of dyads to obtain the confidence intervals (S8 Text).
(XLSX)

S6 Table Pairwise comparisons of RCP values inferred for replicate samples of cultured human ES and iPS cells, and for mouse embryonic cells sampled at various developmental stages. We applied our test for heterogeneity, as described in S5 Text, to assess evidence of significant differences between RCP values inferred from various sample replicates presented by Arand et al. (2015) [17]. A color gradient encodes approximate levels of significance, with dark green indicating non-significant differences, and red indicating highly significant differences.
(XLSX)

S1 Text Deriving the Ratio of Concordance Preference, RCP. (PDF)

S2 Text Comparing RCP values and hemi-preference ratios from HMM. (PDF)

S3 Text Assessing the potential impact of bisulfite-conversion errors. (PDF)

S4 Text Mathematical correction for bisulfite-conversion error. (PDF)

S5 Text Assessment of heterogeneity among replicates and quasi-replicates. (PDF)

S6 Text Pooling data sets across replicates. (PDF)

S7 Text Inferring and comparing RCP without assuming independent sampling of dyads. (PDF)

S8 Text Inferring and comparing RCP with assuming independent sampling of dyads. (PDF)

S9 Text Could spontaneous differentiation of a subset of ES and iPS cells substantially influence the inference of RCP? (PDF)

S10 Text Comparing RCPs of *Dnmt3*-knockout lines with those of wildtype lines. (PDF)
