# Supplementary figures and images for: Epigenetic memory via concordant DNA methylation is inversely correlated to developmental potential of mammalian cells
Source: PLoS Genet. 2017 Nov 6;13(11):e1007060. doi: 10.1371/journal.pgen.1007060 (PMC5690686; doi:10.1371/journal.pgen.1007060)

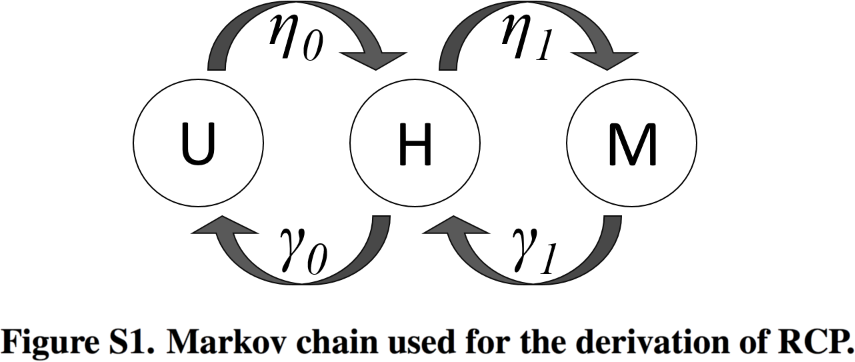

Supplement: S1 Fig — Derivation described in S1 Text. (TIF) [file pgen.1007060.s001.tif]

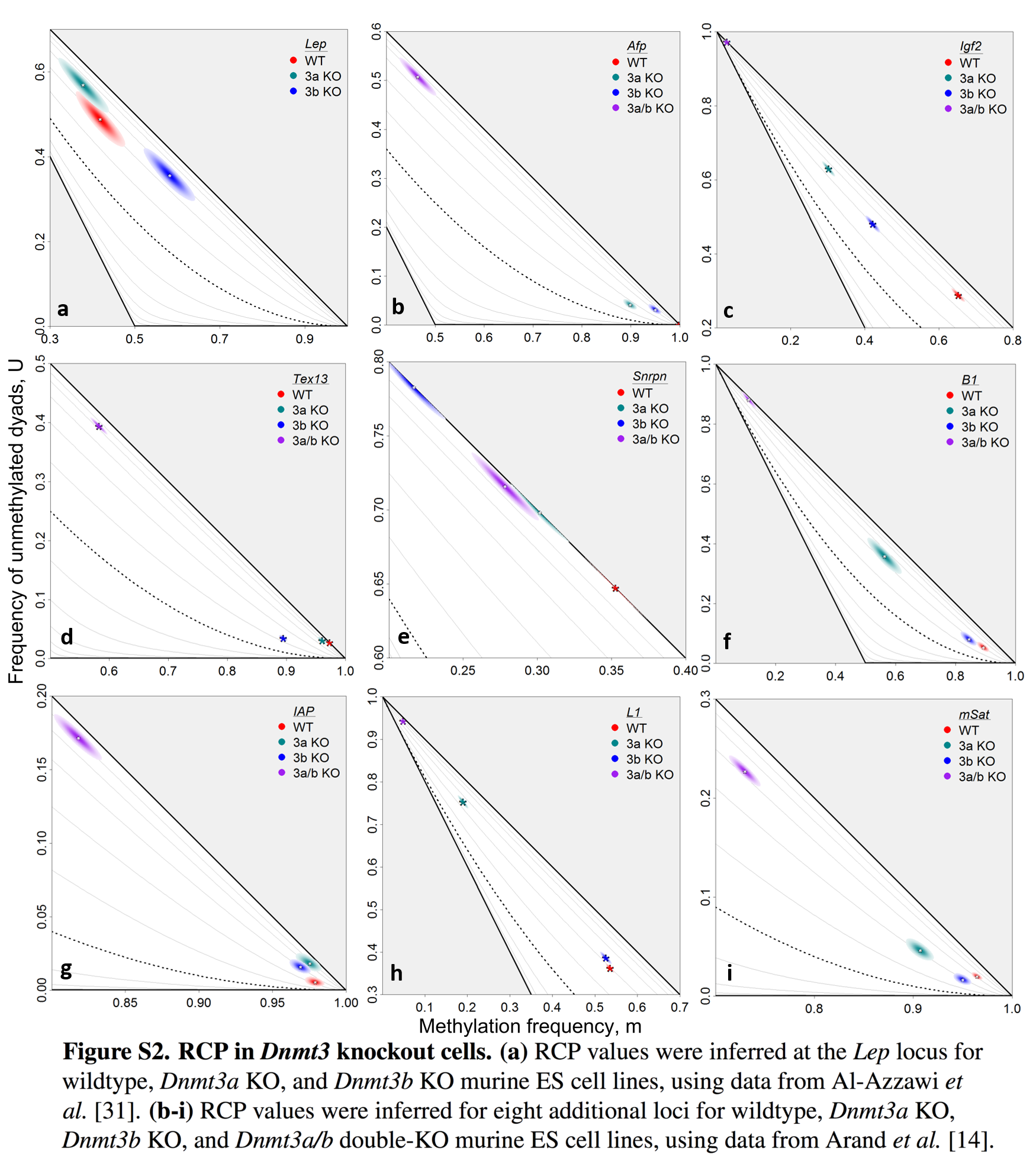

Supplement: S2 Fig — (a) RCP values were inferred at the Lep locus for wildtype, Dnmt3a KO, and Dnmt3b KO murine ES cell lines, using data from Al-Azzawi [32]. (b-i) RCP values were inferred for eight additional loci for wildtype, Dnmt3a KO, Dnmt3b KO, and Dnmt3a/b double-KO murine ES cell lines, using data from Arand et al. [14]. (TIF) [file pgen.1007060.s002.tif]
