# Supplementary material for: Epigenetic memory via concordant DNA methylation is inversely correlated to developmental potential of mammalian cells
Source: PLoS Genet. 2017 Nov 6;13(11):e1007060. doi: 10.1371/journal.pgen.1007060 (PMC5690686; doi:10.1371/journal.pgen.1007060)
Supplement: S2 Table — Entries shown are for patterns published here for the first time. R.E. refers to the restriction enzyme used to create the genomic overhang prior to ligation with a hairpin linker. (PDF) [file pgen.1007060.s004.pdf]

<table>
<tr><th>Organism (cell type)</th><th>Locus</th><th>Accession Number</th><th>R.E.</th><th>Genomic Overhang</th><th>Forward Primer (5’ to 3’)</th><th>Reverse Primer (5’ to 3’)</th><th>PCR Annealing Temp (°C)</th></tr>
<tr><td rowspan="2">Human (cultured fibroblasts)</td><td>FMR1</td><td>181338201
(4820-4900)</td><td>TspRI</td><td>5′....TTCAGTGTT|3′
3′...|--------- 5′</td><td>F1: CRCTCCCAAACCACTTAAA</td><td>R1: TTGAAGAGAGAGGGYGGG</td><td>50 (F1-R1)</td></tr>
<tr><td>LINE1</td><td>M80343.1
(180-323)</td><td>BsmAI</td><td>5′...|---- 3′
3′....TGGT|5′</td><td>F1: CAAAACRAAACATTACCTCACCT</td><td>R1: TTGGGAAGYGTAAGGGGTTAG
R2: GGYGAGGTATTGTTTTATTTG</td><td>49 (F1-R1)
49 (F1-R2)</td></tr>
</table>

Table S2: Hairpin-linkage and PCR conditions for collection of double-stranded DNA methylation patterns. Entries shown are for patterns published here for the first time. R.E. refers to the restriction enzyme used to create the genomic overhang prior to ligation with a hairpin linker.
