# Supplementary material for: Epigenetic memory via concordant DNA methylation is inversely correlated to developmental potential of mammalian cells
Source: PLoS Genet. 2017 Nov 6;13(11):e1007060. doi: 10.1371/journal.pgen.1007060 (PMC5690686; doi:10.1371/journal.pgen.1007060)
Supplement: S1 Text — (PDF) [file pgen.1007060.s009.pdf]

S1 Text: Deriving the Ratio of Concordance Preference, RCP

The goal of RCP is to quantify the extent to which the DNA methylation machineries that gave rise to each data set deviate from random expectations under the binomial distribution, as indicated by an over- or under-abundance of concordant dyads. Here we approach this problem by modeling the formation of concordant and discordant dyads as transitions between unmethylated and hemimethylated dyads and between hemimethylated and fully methylated dyads, without regard to the molecular processes that facilitate these transitions. We then take a mathematical approach to derive an expression for RCP.

We seek the equilibrium frequencies of fully methylated (M), hemimethylated (H), and unmethylated (U), dyads. Consider a continuous-time Markov chain operating on the probability distribution of the dyads $\langle M, H, U \rangle$:

$$\frac{d}{dt}\begin{pmatrix} M \\ H \\ U \end{pmatrix} = \begin{pmatrix} -2\gamma_1 & \eta_1 & 0 \\ 2\gamma_1 & -\eta_1 - \gamma_0 & 2\eta_0 \\ 0 & \gamma_0 & -2\eta_0 \end{pmatrix} \begin{pmatrix} M \\ H \\ U \end{pmatrix} \tag{1}$$

where η's and γ's represent the rates of methylation addition and removal, respectively, as shown in S1 Fig.

We define RCP as the ratio between β_c, the rate of dyad transitions yielding concordant dyads, and β_d, the rate of dyad transitions yielding discordant dyads. We thus define $\beta_c = \sqrt{\eta_1\gamma_0}$, the geometric mean of the methyl-addition and methyl-removal rates yielding concordant dyads. Likewise, we define $\beta_d = \sqrt{\eta_0\gamma_1}$.

We can then solve for the steady state distribution for the Markov chain in Equation (1) to arrive at the ratio. We can also express it in terms of m and U, as shown below.

$$\text{RCP} := \frac{\beta_c}{\beta_d} = \frac{\sqrt{\eta_1\gamma_0}}{\sqrt{\eta_0\gamma_1}} = \sqrt{\frac{4MU}{H^2}} = \frac{\sqrt{U(U+2m-1)}}{1-U-m} \tag{2}$$

This formulation of RCP does not require the assumption that methylation frequency is constant over time. Here, "steady state" refers to the dyad frequencies expected under a given system of methylation processes, regardless of whether the methylation frequency is constant over rounds of cell division.

It is notable that RCP^2 is $4MU/H^2$, which is expected to equal 1 under the Hardy-Weinberg equilibrium [21], if dyad frequencies are considered as genotype frequencies of a gene with two alleles. Following this, RCP can be considered as a measure of deviation from the null equilibrium.

RCP is therefore a metric for the degree to which the system of methylation processes prefers concordance (RCP > 1), discordance (RCP < 1), or, possibly, exhibits no preference in either direction (RCP $= 1$). If we set RCP $= 1$ and solve this expression for U, we find that $U = (1-m)^2$. This is consistent with the expectation under the binomial distribution that RCP will be 1 when there is no preference for either concordance or discordance. As RCP approaches ∞, U approaches $1 - m$. Setting RCP $= 0$ results in two solutions: $U = 1 - 2m$ and $U = 0$. These solutions are congruent with the boundaries that define the space of (m, U) as given in "Ratio of Concordance Preference is Defined for All Possible Configurations of Methylation at Symmetric Nucleotide Motifs" of the main text, and in Fig 1.
