# Supplementary material for: Epigenetic memory via concordant DNA methylation is inversely correlated to developmental potential of mammalian cells
Source: PLoS Genet. 2017 Nov 6;13(11):e1007060. doi: 10.1371/journal.pgen.1007060 (PMC5690686; doi:10.1371/journal.pgen.1007060)
Supplement: S2 Text — (PDF) [file pgen.1007060.s010.pdf]

S2 Text: Comparing RCP Values and Hemi-Preference Ratios from HMM

"Hemi-preference ratio", a parameter inferred under our earlier analysis with a hidden-Markov model (HMM) [24], evaluates the preference of a given DNA methyltransferase for acting at hemimethylated as compared to unmethylated dyads [11], and thereby measures its preference for creating concordant dyads. Fu *et al.* [24,28] calculated this ratio for DNMT1, a mammalian maintenance methyltransferase. Because RCP measures the concordance preference of the entire ensemble of enzymes that give rise to methylation patterns, the hemi-preference ratio of a given enzyme and the RCP value inferred from the same data set are expected to have good agreement if that enzyme is the primary actor. The congruence between these two metrics is expected to decline with increasing contributions from other enzymes.

Three of the four data sets analyzed previously under HMM showed very good agreement between the RCP values we infer here and the hemi-preference ratios previously inferred for the maintenance methyltransferase DNMT1: 58.0 vs. 58 for *FMR1*, 13.3 vs. 15 for *G6PD*, and 89.1 vs. 94 for *LEP* [24] (S1 Table). The close correspondence between these values indicates that for these loci in leukocytes, methylation dynamics are driven primarily by conservative, maintenance-type processes such as accomplished by DNMT1, and that neither active demethylation nor *de novo* processes have a substantial role. Furthermore, when there is such a correspondence, RCP strongly suggests that the mechanistic assumptions made for the enzymatic model hold for that data set.

A large discrepancy, on the other hand, may suggest shortcomings of the mechanistic model. The fourth data set that had been analyzed under HMM, *LEP* in human adipose tissue, had an inferred RCP value of 34, well within the range of RCPs inferred for other data sets from single-copy loci including *LEP* in leukocytes (Fig 1a; S1 Table). This RCP value was, however, more than eighteen-fold lower than the DNMT1 hemi-preference ratio estimate of 628 that we obtained under the earlier HMM approach (S1 Table). What might account for this discrepancy? A hemi-preference ratio of 628 is unrealistically high, even for a maintenance enzyme, compared to the hemi-preference ratios inferred in other data sets, including those from methylation patterns established by DNMT1 *in vitro* [11]. This could reflect the inability of the HMM to yield a reasonable estimate for a data set impacted by demethylation, a process that was not considered in the HMM design. This lack of correspondence between HMM and RCP estimates is consistent with the possibility that active removal of methylation has a heightened role at loci with temporally variable transcription levels, and may reflect the role of the *LEP* locus as a sentinel of adipose but not blood [29].
