# Supplementary material for: Epigenetic memory via concordant DNA methylation is inversely correlated to developmental potential of mammalian cells
Source: PLoS Genet. 2017 Nov 6;13(11):e1007060. doi: 10.1371/journal.pgen.1007060 (PMC5690686; doi:10.1371/journal.pgen.1007060)
Supplement: S3 Text — (PDF) [file pgen.1007060.s011.pdf]

S3 Text: Assessing the Potential Impact of Bisulfite-Conversion Errors

Two classes of error that occur during bisulfite conversion can lead to misinterpretation of cytosine methylation states. Failure to convert unmethylated cytosines to uracil occurs at rate b, and can result in overestimation of methylation frequency. Inappropriate conversion of methylated cytosines to thymine, first noted by Shiraishi and Hayatsu [50], occurs at rate c, and can result in underestimation of methylation frequency. We have reported previously that the rates of failed and inappropriate conversion depend strongly on the chemical and thermal conditions of bisulfite conversion [49]. In particular, we found that bisulfite treatment prolonged beyond that required to attain complete or nearly complete conversion of unmethylated cytosines can yield high rates of inappropriate conversion. Historically, conversion protocols have been designed to minimize the failed-conversion rate, with little or no attention to the rate of inappropriate conversion events. Thus, while both classes of error can alter parameters used to infer RCP — the methylation frequency, m, and the unmethylated dyad frequency, U — errors arising through inappropriate conversion are likely of more substantial impact.

How severely can conversion error impact RCP? And to what extent does its potential impact depend on the true methylation frequency of the target sequences? Densely methylated sequences contain a large number of fully methylated dyads, such that the most likely conversion error is inappropriate conversion yielding apparent hemimethylated dyads. Such events elevate the apparent level of discordance in a given data set and artifactually depress the inferred RCP values. To quantify this potential impact, we calculated how RCP would be altered by a single artifactual hemimethylated dyad introduced by inappropriate conversion, assuming that in its true state the relevant data set had one unmethylated dyad, one hemimethylated dyad, and 98 methylated dyads. The inappropriate conversion of one cytosine among 98 dyads whose true state is fully methylated is close to the number expected for an inappropriate conversion rate of about 0.5%, a conservative estimate of this error rate [49]. For this hypothetical data set, the introduction of a lone artifactual hemimethylated dyad by an inappropriate conversion event reduces RCP from about 20 to about 10, i.e., by a factor of 2. Thus, in the absence of mathematical corrections of the sort implemented here, even very low levels of inappropriate conversion can severely impact inference of RCP.

Sparsely methylated sequences contain a large number of unmethylated cytosines that are potential targets for failed conversion. We calculated that for such sequences the impact of conversion error depends on whether most unmethylated cytosines are in hemimethylated or in unmethylated dyads. For example, when most unmethylated cytosines in a sparsely methylated sequence occur in hemimethylated dyads, the level of discordance is already high, such that the addition of an artifactual hemimethylated dyad by failed conversion only slightly decreases RCP. By contrast, when most unmethylated cytosines are in unmethylated dyads, production of an artifactual hemimethyated dyad by failed conversion reduces RCP by a factor of two, as illustrated in the previous paragraph. Mathematical correction for conversion error is therefore critical, not only because error has potentially large impacts on RCP values, but also because the magnitude of these impacts differs among data sets, with the potential either to magnify or to dampen variation among them.

References

50. Shiraishi M, Hayatsu H. High-speed conversion of cytosine to uracil in bisulfite genomic sequencing analysis of DNA methylation. DNA Research. 2004;11(6):409–415.
