# Supplementary material for: Epigenetic memory via concordant DNA methylation is inversely correlated to developmental potential of mammalian cells
Source: PLoS Genet. 2017 Nov 6;13(11):e1007060. doi: 10.1371/journal.pgen.1007060 (PMC5690686; doi:10.1371/journal.pgen.1007060)
Supplement: S4 Text — (PDF) [file pgen.1007060.s012.pdf]

S4 Text: Mathematical Correction for Bisulfite-Conversion Error

To account for conversion errors occurring at known rates, we express the observed dyad frequencies, M_{fobs}, H_{fobs}, and U_{fobs} , as functions of the true dyad frequencies M_t, H_t, and U_t, that would have been observed had conversion error not occurred:

$$\begin{aligned} M_{fobs}(M_t, H_t, U_t, b, c) &= M_t\,(1-c)^2 + H_t\,b\,(1-c) + U_t\,b^2 \\ H_{fobs}(M_t, H_t, U_t, b, c) &= 2\,M_t\,c\,(1-c) + H_t\,(1-b)\,(1-c) + 2\,U_t\,b\,(1-b) \\ U_{fobs}(M_t, H_t, U_t, b, c) &= M_t\,c^2 + H_t\,(1-b)c + U_t\,(1-b)^2 \end{aligned} \quad (1)$$

In cases where the mathematical correction yielded negative counts for one or two dyad types, the negative counts were redistributed to the remaining dyad types in proportion to the original dyad counts, such that no dyad counts were negative after correction.

For each of the data sets reported here, DNAs were converted under one of two conditions: low molarity-temperature (LowMT), with failed-conversion rate 0.0030 and inappropriate conversion rate 0.031, and high molarity-temperature (HighMT), with failed-conversion rate 0.0086 and inappropriate-conversion rate 0.017 [49]. Error rates used in analysis of published data from other groups are given in S4 Table (Arand *et al.* [14,17]) and S5 Table (Zhao *et al.* [15]). Using these rates, observed dyad frequencies, and Equation 1, true dyad frequencies can be inferred.
