# Supplementary material for: Epigenetic memory via concordant DNA methylation is inversely correlated to developmental potential of mammalian cells
Source: PLoS Genet. 2017 Nov 6;13(11):e1007060. doi: 10.1371/journal.pgen.1007060 (PMC5690686; doi:10.1371/journal.pgen.1007060)
Supplement: S5 Text — (PDF) [file pgen.1007060.s013.pdf]

S5 Text: Assessment of Heterogeneity Among Replicates and Quasi-Replicates

To ask about possible heterogeneity among RCP values inferred for replicates — for example, for individual developmental stages as investigated by Arand *et al.* [17] — and for quasi-replicates — for example, for multiple cell lines at similar cell-culture and differentiation conditions — we utilized pairwise, two-tailed permutation tests.

These calculations revealed an intriguing pattern. There was no evidence of heterogeneity among samples of cultured human ES and iPS cells ($p > 0.29$, pairwise two-tailed PTs for the six cultured stem cell lines at the *L1* locus; p-values summarized in S6 Table; Fig 3). By contrast, several pairwise comparisons of mouse embryonic cells sampled independently for a given developmental stage yielded evidence of significant differences in RCP (p-values summarized in S6 Table). Notably, however, most of the significant heterogeneity observed was for cells in pronuclear stages, at which data collection at identical stages is made difficult by rapid developmental transitions. In contrast, there was evidence of only limited heterogeneity among totipotent and pluripotent cells between the 2-cell stage and the 3.5-dpc stage. Similarly, when experimental interventions were taken to prevent DNA replication or methylation (i.e. aphidicolin treatment at PN4/5 stages and SAMase treatment just after fertilization and before the first round of DNA replication), no evidence of heterogeneity was observed. Cell culture techniques are focused on minimizing opportunities for developmental differences to arise among cells, and could account for the observation of no heterogeneity among the cultured human ES and iPS cells.

Given the low levels of heterogeneity observed and the fact that some level of heterogeneity is inevitable in cells undergoing rapid epigenetic transitions, we pooled several sets of replicates for analysis, as described in S6 Text (Fig 6).
