# Supplementary material for: Epigenetic memory via concordant DNA methylation is inversely correlated to developmental potential of mammalian cells
Source: PLoS Genet. 2017 Nov 6;13(11):e1007060. doi: 10.1371/journal.pgen.1007060 (PMC5690686; doi:10.1371/journal.pgen.1007060)
Supplement: S6 Text — (PDF) [file pgen.1007060.s014.pdf]

S6 Text: Pooling Data Sets Across Replicates

In some instances, multiple independent replicates were collected and used to assess RCP for cells under a given biological condition — for example, multiple embryos at a given pronuclear stage (Fig 6). In these cases, the sequence-level dyad counts of individual replicate data sets were corrected for conversion errors independently before the replicate data sets were pooled.

When using likelihood methods to quantify and to assess pooled data sets – thus assuming independence of dyads (S8 Text) – we explicitly allow m to vary across replicates while estimating a single RCP value in the likelihood-maximization process. When using bootstrap methods (S7 Text), as we do whenever individual-molecule data are available, we do not explicitly account for the possibility that m may vary across the replicate data sets. This may result in a slight enlargement of confidence intervals in the bootstrap process.
