# Supplementary material for: Epigenetic memory via concordant DNA methylation is inversely correlated to developmental potential of mammalian cells
Source: PLoS Genet. 2017 Nov 6;13(11):e1007060. doi: 10.1371/journal.pgen.1007060 (PMC5690686; doi:10.1371/journal.pgen.1007060)
Supplement: S7 Text — (PDF) [file pgen.1007060.s015.pdf]

S7 Text: Inferring and Comparing RCP Without Assuming Independent Sampling of Dyads

CpG dyads typically are not sampled individually, but instead as members of sequence reads that can contain from a few to many neighboring dyads. Often, there is correlation among the methylation states of these neighboring dyads. The processivity of the DNA methyltransferases, especially Dnmt1, is a substantial contributor to these correlations. As the mean number of dyads per read increases, so too does the potential for dyad-dyad correlation to undermine the accuracy of confidence intervals inferred under the assumption of independent sampling of dyads.

Of the three groups of data we analyzed here, one — Zhao *et al.* (2014) [15] — consists of Illumina paired-end reads. These reads span 40 to 60 genomic nucleotides, and so are much shorter than those generated by our methods and those of Arand *et al.*. On average, each of the read pairs in Zhao *et al.* provides methylation data for only 1.2 CpG dyads. As this CpG count is only slightly greater than the condition of 1 CpG dyad per read that would provide for complete independence among sampled dyads, we anticipate that correlation among methylation states of dyads ascertained on a given read by Zhao *et al.* will have only a minor impact on sampling. The subset of these reads that derive from CpG-rich CpG Islands do contain more CpGs, likely up to 3 [51]. However, this sequence class contributes only 1.2% of CpG dyads in the "All" CpG dataset. Reads for our data have as many as 22 CpG dyads; mean dyad counts for data from Arand et al. [14,17] are intermediate between our data and those of Zhao *et al.* (2014) [15].

For our own data and those of Arand *et al.* [14,17], we used an approach that, at slightly higher computational cost, models and seeks to account for the potential impacts of dyad-dyad correlations. Our data yielded moderately larger confidence intervals under the bootstrapping approach as compared to under the likelihood approach with the assumption of independent sampling of dyads. By contrast, the data from Arand *et al.* [14,17] yielded almost identical confidence intervals whether without or with the assumption of independence. In view of the even smaller mean number of dyads per read in the data of Zhao *et al.* (2014) [15], we chose to make the assumption that dyads were sampled independently in their data. Although it is possible that two or more reads originated from nearby regions of a single molecule and thus have dyad-dyad dependence, we assumed that the effect of such occurrences, if at all present, is very small, given the large amount of starting material.

The first of the two methods is described in this section, and the second in the next section, S8 Text.

Inferring RCP point estimates and confidence intervals. For our own data and those of Arand *et al.*, we used a bootstrapping approach to model the uncertainty in RCP values introduced by possible within-sequence correlations in methylation states, and to make point estimate and confidence-interval inferences that account for this uncertainty.

For each data set of n sequences, we sampled n sequences with replacement, $B =$ 2,000,000 times. For each of these bootstrapped sets, RCP was calculated by summing the M, H, and U dyad counts for all of the resampled molecules and using Equation 2 in S1 Text. We inferred the true distribution of RCP for a given observed data set from the distribution of these B bootstrapped RCP values. It was clear from the resulting distributions that RCP is a biased estimator, as many of these distributions had longer right tails than left.

The simplest, and potentially misleading, approach for inference of point estimates and construction of confidence intervals from bootstrap distributions is to assume normality, and

then to exclude right and left tails at the intended level of confidence. Efron and DiCiccio (1996) [52] commented that, for biased estimators, this approach can lead to inference of inappropriately exclusive limits at the long-tailed end of the distribution, and inappropriately inclusive limits at the short-tailed side.

To address this problem, we applied Efron and Diccicio's "bias-corrected and accelerated" (BCa) method. Under the BCa method, the cumulative-density function observed for the distribution of bootstrap replicates is compared to that expected under normality. Bias-corrected point estimates are then inferred as the 50th-percentile values in the BCa- corrected distributions. Similarly, critical points for intervals of a given confidence level are inferred from the values at the relevant percentiles of the distribution of bootstrapped values.

Because methylation states are predicted to be correlated across dyads within a molecule, but not across molecules, we resample at the level of molecules. Furthermore, as noted above, our molecular-barcoding procedures enable exclusion of redundant reads, such that each methylation pattern in our resulting data set is known to derive from a unique molecule in the original sample. Moreover, it appears that molecules are sampled without bias due to methylation: in all eight data sets from murine DNA methyltransferase knockout lines (Fig 4) and all six data sets from wildtype human cells (Fig 3) there was no evidence of correlation between methylation frequency and RCP. Thus, it is reasonable to consider sampled molecules as independent and identically distributed draws from a population.

For each sampled molecule we derive a vector of values, (M_i, H_i, U_i, n_i), where n_i is the number of dyads. These are the vectors we resample in our procedure. We see that RCP can be written, using this vector, as

$$RCP = \frac{\sqrt{4\bar{M}\bar{U}}}{\bar{H}} \tag{1}$$

where $\bar{M} = \frac{\sum n_i M_i}{\sum n_i}$, $\bar{H} = \frac{\sum n_i H_i}{\sum n_i}$ and $\bar{U} = \frac{\sum n_i U_i}{\sum n_i}$. Now considering the vector $(n_i M_i, n_i H_i, n_i U_i, n_i)$ and, noting that the ratio of sums can be written as a ratio of means, we see that this falls directly under the "smooth function of means" framework introduced in [53] for the standard bootstrap and applied in [54] to the BCa bootstrap. From results in Section 6 of [54], we conclude that our bootstrap procedure gives approximate confidence intervals with asympotically correct coverage.

Assessing whether a data set has RCP value greater than 1. If methyl groups are placed completely at random — that is, with preference for neither concordance nor discordance — RCP is expected to be 1. In a previous report, Shipony *et al.* [16] interpreted their data to indicate that methyl groups are, indeed, placed essentially at random in undifferentiated cells. As there is very little evidence in any of our data sets to indicate possible preference for discordance, we opted to perform a one-tailed bootstrap test (BT), asking for the probability that our data sets do not have RCP values greater than 1.

To do so, we first calculated RCP point estimates for 200,000 bootstrap replicates and performed the BCa correction, using the method described above, and then calculated the approximate p-value as the fraction of those point estimates that were less than or equal to RCP of 1. For example, from the finding that only 20 of the 200,000 bootstrap replicates yielded an RCP point estimate less than 1, we would conclude that RCP is significantly greater than 1 with an approximate p-value of 0.0001. Note that we shifted from the 2,000,000 bootstrap draws noted above to the 200,000 reported here upon finding only trivial differences between p-values derived under these two approaches.

Assessing whether RCP values differ significantly between data sets. A key goal of our

study is to assess possible evidence for RCP differences between data sets. For example, we ask whether RCP for a given cell type differs between samples collected under differentiating as compared to non-differentiating conditions.

To compare two data sets that can be modeled by the same null distribution, as is the case for most comparisons we make here, we performed a permutation test (PT). For comparisons in which a shared null distribution cannot be established (such as would be the case if the two data sets were from different loci and thus had different numbers and locations of dyads), we used a bootstrap test (BT). We describe these two methods below.

To compute the significance of observed differences between RCP values for Data Sample A and Sample B, which can share a null distribution, we used permutation to compute the null distribution of ordered differences (for example, $\text{RCP}(\text{Sample A}) - \text{RCP}(\text{Sample B})$) expected under the null assumption that the sequences in the two sets were drawn from a single population. To do this, we pooled sequences from Sample A and Sample B and then repeatedly drew from that pool, without replacement, to generate, at random, versions of Sample A and Sample B with sequence counts equal to those of the observed data. For each pair of randomly generated sets, we calculated the difference between their RCP point estimates (θ^*) , and obtained the distribution of RCP-difference values under the null hypothesis. For one-tailed tests, for example, to ask whether one data set has RCP value greater than the RCP value for another, we took the proportion of the distribution greater than the difference of the point estimates ($\hat{\theta}$) as the p value. For two-tailed/equal-tailed tests, for example, to ask whether RCP values for two data sets differ significantly, we first calculated the proportion of permuted differences smaller than $\hat{\theta}$. We then calculated the proportion of permuted differences greater than $\hat{\theta}$. We took the twice the lesser of these two values as the p-value for observing a difference this great in the event that the sequences in the two data sets were, in reality, drawn from the same distribution.

To compute the significance of observed differences between RCP values for two samples with different generating distributions, we used a bootstrap-based comparison test. Instead of pooling the data sets to form a single population of molecules, we bootstrapped a single RCP value from each of the two separate populations of molecules and computed the difference. We repeatedly sampled this difference to draw a bootstrap distribution of the difference in RCP values. For one-tailed tests, with which we examine directional differences, we determined the p-value as the proportion of bootstrap-difference samples to the left of 0. For two-tailed tests, with which we can detect differences in any direction, we determined the p-value as twice the smaller proportion of the bootstrap difference samples on either side of 0.

Defining the approximate 95% confidence region for a data set in the (m, U) space, without assuming independent sampling of dyads. Using the methods described above, we generated 2,000,000 bootstrap samples of each data set. Instead of estimating the RCP value for each of the bootstrap samples, we calculated m and U. We constructed the two-dimensional confidence region for the two parameters for plotting using `ci2d` function in the `gplots` R package.

References

51. Glass JL, Thompson RF, Khulan B, Figueroa ME, Olivier EN, Oakley EJ, et al. CG dinucleotide clustering is a species-specific property of the genome. Nucleic acids research. 2007;35(20):6798–6807.

52. DiCiccio TJ, Efron B. Bootstrap confidence intervals. Statistical science. 1996;p.

189–212.

53. Hall P. The Bootstrap and Edgeworth Expansion. 1992. Springer, New York;.

54. DiCiccio TJ, Efron B. Bootstrap confidence intervals. Statistical science. 1996;p. 189–212.
