# Supplementary material for: Epigenetic memory via concordant DNA methylation is inversely correlated to developmental potential of mammalian cells
Source: PLoS Genet. 2017 Nov 6;13(11):e1007060. doi: 10.1371/journal.pgen.1007060 (PMC5690686; doi:10.1371/journal.pgen.1007060)
Supplement: S8 Text — (PDF) [file pgen.1007060.s016.pdf]

S8 Text: Inferring and Comparing RCP With Assuming Independent Sampling of Dyads

Calculating the likelihood of proposed true dyad frequencies, M_t, H_t, and U_t, given the observed dyad counts, $M_{c\,obs}$, $H_{c\,obs}$, and $U_{c\,obs}$. Failed- and inappropriate-conversion events create observed dyad frequencies that differ from true dyad frequencies. If the rates of these two types of error are known, the likelihood of a set of proposed true frequencies — M_t, H_t, and U_t — can be calculated as follows, given the observed dyad counts — $M_{c\,obs}$, $H_{c\,obs}$, and $U_{c\,obs}$ — and Equation 1 of S4 Text. M_{fobs}, H_{fobs}, and U_{fobs}, which indicate the observed frequencies of the dyads, can be easily calculated from the dyad counts.

$$\begin{aligned}\mathcal{L}(M_t, H_t, U_t \mid M_{c\,obs}, H_{c\,obs}, U_{c\,obs}, b, c) = C \times M_{fobs}(c, b, M_t, H_t, U_t)^{M_{c\,obs}} \\ \times H_{fobs}(c, b, M_t, H_t, U_t)^{H_{c\,obs}} \\ \times U_{fobs}(c, b, M_t, H_t, U_t)^{U_{c\,obs}} \\ \text{with multinomial coefficient } C = \frac{(M_{c\,obs} + H_{c\,obs} + U_{c\,obs})!}{M_{c\,obs}!\; H_{c\,obs}!\; U_{c\,obs}!}\end{aligned} \quad (1)$$

Inferring RCP point estimates and confidence intervals. The RCP point estimate of a data set is calculated directly from the conversion-error-corrected observed dyad frequencies. We determine the approximate 95% confidence interval for RCP as the interval that includes all values of RCP for which the natural log likelihood lies within $\chi^2_{0.95;\, df=1} = 1.92$ units of the maximum natural log likelihood point estimate [55].

Although bias is just as much a concern with the assumption of independent sampling of dyads, we did not perform a bias correction for the data from Zhao *et al.* [15], because without bootstrapping, we lacked a simple method for bias estimation. Nonetheless, our analyses of other data sets suggest that most of these samples are likely not severely affected by bias. From bootstrapping of smaller data sets collected by our lab and by Arand *et al.* [14,17], we have observed that the asymmetry in the distribution is small in the lower ranges of RCP (1∼10), but greater as RCP increases. Therefore, bias is likely to be small for most samples presented by Zhao *et al.*, for which RCP point estimates rarely exceed 10. For data sets presented by Zhao *et al.* that yielded RCP estimates greater than 10, biases are unlikely to affect the general conclusion of methylation behavior characterized by strong preference for concordance.

Assessing whether RCP values differ significantly between two data sets. To compare the RCP values between two data sets while assuming independence among all dyads, we can use a likelihood approach, comparing a model in which two true RCP values are required to described the two data sets to an alternate model in which both data sets can be explained by a single RCP value. We implemented this test, maximum likelihood comparison test (MLCT), as follows:

Solving for U in Equation 2 of S1 Text gives:

$$U(m, \text{RCP}) = 1 - m - \frac{1}{2}\left(\frac{1 - \sqrt{1 - 4m(1-m)\left(1 - \text{RCP}^2\right)}}{1 - \text{RCP}^2}\right) \quad (2)$$

Using this, we can also define $M(m, \text{RCP})$ and $H(m, \text{RCP})$. Modifying Equation 1 of S4 Text, we can derive the expressions for $M_{fobs}(c, b, m, \text{RCP})$, $H_{fobs}(c, b, m, \text{RCP})$, and

$U_{fobs}(c, b, m, \text{RCP})$. We then can rewrite Equation 1, such that the parameters are c, b, m, and RCP:

$$\begin{aligned}\mathcal{L}_{one\ set}(M_{fobs}, H_{fobs}, U_{fobs} \mid c, b, m, \text{RCP}) = C \times M_{fobs}(c, b, m, \text{RCP})^{M_{cobs}} \\ \times H_{fobs}(c, b, m, \text{RCP})^{H_{cobs}} \\ \times U_{fobs}(c, b, m, \text{RCP})^{U_{cobs}}\end{aligned} \tag{3}$$

We then employ a likelihood-ratio test to quantify the fit of an alternative model relative to the null. In the null model, which has three variable parameters, m^1_{null}, m^2_{null}, and RCP_{null}, one value of RCP explains both data sets. Using Equation 3:

$$\begin{aligned}\mathcal{L}_{null}(M^1_{fobs}, H^1_{fobs}, U^1_{fobs}, M^2_{fobs}, H^2_{fobs}, U^2_{fobs} \mid c^1, b^1, m^1_{null}, c^2, b^2, m^2_{null}, \text{RCP}) \\ = L_{one\ set}(M^1_{fobs}, H^1_{fobs}, U^1_{fobs} \mid c^1, b^1, m^1_{null}, \text{RCP}_{null}) \\ \times L_{one\ set}(M^2_{fobs}, H^2_{fobs}, U^2_{fobs} \mid c^2, b^2, m^2_{null}, \text{RCP}_{null}).\end{aligned} \tag{4}$$

The alternate model, which has four variable parameters, m^1_{alt}, m^2_{alt}, RCP^1_{alt}, and RCP^2_{alt}, has two values of RCP, one for each data set.

$$\begin{aligned}\mathcal{L}_{alt}(M^1_{fobs}, H^1_{fobs}, U^1_{fobs}, M^2_{fobs}, H^2_{fobs}, U^2_{fobs} \mid c^1, b^1, m^1_{alt}, \text{RCP}^1_{alt}, c^2, b^2, m^2_{alt}, \text{RCP}^2_{alt}) \\ = L_{one\ set}(M^1_{fobs}, H^1_{fobs}, U^1_{fobs} \mid c^1, b^1, m^1_{alt}, \text{RCP}^1_{alt}) \\ \times L_{one\ set}(M^2_{fobs}, H^2_{fobs}, U^2_{fobs} \mid c^2, b^2, m^2_{alt}, \text{RCP}^2_{alt}).\end{aligned} \tag{5}$$

Computing the ratio of the maximum likelihoods for the null and alternate models, we can calculate the test statistic, D:

$$D = -2\, ln\left(\frac{\mathcal{L}_{null}(\hat{m}^1_{null}, \hat{m}^2_{null}, \hat{\text{RCP}}_{null})}{\mathcal{L}_{alt}(\hat{m}^1_{alt}, \hat{\text{RCP}}^1_{alt}, \hat{m}^2_{alt}, \hat{\text{RCP}}^2_{alt})}\right) \tag{6}$$

Under the assumption of large sample of dyads, D is approximately χ^2 distributed with 1 degree of freedom.

Assessing whether a data set has RCP value greater than 1. We again take a likelihood-based approach as we did in the section above, and define a modified maximum likelihood comparison test (MLCT) as follows. Here, the null model states that the system operates under the specified RCP value. The alternate model states that the system operates under another RCP value.

$$\begin{aligned}\mathcal{L}_{null}(\text{RCP}_{\text{specified}}, m_1, ..., m_n \mid M_{c\,obs\,1}, H_{c\,obs\,1}, U_{c\,obs\,1}, b_1, c_1, ..., M_{c\,obs\,n}, H_{c\,obs\,n}, U_{c\,obs\,n}, b_n, c_n) \\ = \prod_i^n \mathcal{L}(m_i, \text{RCP}_{\text{specified}} \mid M_{c\,obs\,i}, H_{c\,obs\,i}, U_{c\,obs\,i}, b_i, c_i)\end{aligned} \tag{7}$$

$$\begin{aligned}\mathcal{L}_{alt}(\text{RCP}_{alt}, m_1, ..., m_n \mid M_{c\,obs\,1}, H_{c\,obs\,1}, U_{c\,obs\,1}, b_1, c_1, ..., M_{c\,obs\,n}, H_{c\,obs\,n}, U_{c\,obs\,n}, b_n, c_n) \\ = \prod_i^n \mathcal{L}(m_i, \text{RCP}_{alt} \mid M_{c\,obs\,i}, H_{c\,obs\,i}, U_{c\,obs\,i}, b_i, c_i)\end{aligned} \tag{8}$$

We treat the two likelihood functions differently in maximizing them. For the alternate model, both n values of m and RCP_{alt} are parameters for maximization. For the null model, the RCP value is specified and thus fixed; only the n values of m are parameters for maximization.

$$D = -2\,ln \left(\frac{\mathcal{L}_{null}(\text{RCP}_{\text{specified}}, \hat{m}_1, ..., \hat{m}_n \mid ...)}{\mathcal{L}_{alt}(\hat{\text{RCP}}_{alt}, \hat{m}_1, ..., \hat{m}_n \mid ...)} \right) \tag{9}$$

Under the assumption of a large sample of dyads, D is approximately χ^2 distributed with 1 degree of freedom.

Defining the approximate 95% confidence region for a data set in the (m, U) space. We determine the approximate 95% confidence region in the space of two parameters — here m and U — as the region that includes all proposed pairs of parameter values for which the natural log likelihood lies within $\chi^2_{0.95;\, df=2} = 3.00$ units of the maximum natural log likelihood point estimate [55].

References

55. Meeker WQ, Escobar LA. Teaching about approximate confidence regions based on maximum likelihood estimation. The American Statistician. 1995;49(1):48–53.
