# Supplementary material for: Epigenetic memory via concordant DNA methylation is inversely correlated to developmental potential of mammalian cells
Source: PLoS Genet. 2017 Nov 6;13(11):e1007060. doi: 10.1371/journal.pgen.1007060 (PMC5690686; doi:10.1371/journal.pgen.1007060)
Supplement: S9 Text — (PDF) [file pgen.1007060.s017.pdf]

S9 Text: Could Spontaneous Differentiation of a Subset of ES and iPS Cells Substantially Influence the Inference of RCP?

One possible explanation for the inference of conservative processes operating in cultured, undifferentiated cells is that these cells may in reality be a mixture of differentiated and undifferentiated cells. We calculated how large the differentiated subpopulation would need to be under this scenario to yield the observed RCP values for the ES and iPS cells, given that the subpopulation operated with the RCP inferred for the corresponding differentiated cells. The remainder of the population was assumed to operate at RCP of 1, per the alternate hypothesis. We allowed specification of m for each of the two populations.

Let p_1 and p_2 represent the proportions of the two putative subpopulations that we wish to estimate, such that $p_1 + p_2 = 1$. We start with RCP and m for each of the two subpopulations, which we denote by RCP_1, RCP_2, m_1, and m_2. We can find U_1 and U_2 using the RCP and m values. We have the following set of equations:

$$p_1 m_1 + p_2 m_2 = m_{\text{overall}}$$
$$p_1 U_1 + p_2 U_2 = U_{\text{overall}}$$

Using these equations and the observed overall RCP value, and the expression for RCP given m and U (Equation 2 of S1 Text), we can find p_1 and p_2.

Assuming that m of the undifferentiated subpopulation is at least 0.2, we found that over half of the cells would need to be differentiated if methylation in the remaining undifferentiated cells operates at RCP of 1 (calculated using data sets presented in Fig 5a). Morphological inspection of the cultured human stem cells did not suggest the presence of such a substantial subpopulation of differentiated cells. Moreover, comparison of RCP for totipotent and pluripotent stem cells from murine embryos revealed values very similar to those inferred for all cultured ES and iPS cell lines that we examined, corroborating the interpretation that preference for concordance is present in stem cells.
