# Supplementary material for: Epigenetic memory via concordant DNA methylation is inversely correlated to developmental potential of mammalian cells
Source: PLoS Genet. 2017 Nov 6;13(11):e1007060. doi: 10.1371/journal.pgen.1007060 (PMC5690686; doi:10.1371/journal.pgen.1007060)
Supplement: S10 Text — (PDF) [file pgen.1007060.s018.pdf]

S10 Text: Comparing RCPs of *Dnmt3*-Knockout Lines with Those of Wildtype Lines

The relative contributions of conservative processes are expected to be more substantial when the fraction of methylation events achieved through maintenance-type activity is elevated through loss of one or both of the *de novo* enzymes. Indeed, at the *Lep* locus, we inferred RCP values of 8.11 for *Dmnt3a* KO cells, a value significantly higher than that for wildtype cells ($p = 0.046$, two-tailed PT; S2 Fig a; S3 Table). Although the point estimate was higher for *Dmnt3b* KO cells as well, the difference was not significant ($p = 0.11$, two-tailed PT).

Examination of data from Arand *et al.* [14] for a broader set of loci in cell lines with knockouts at one or both of the Dnmt3s, however, revealed a more complex role for these enzymes in shaping methylation concordance. Overall methylation levels were diminished in the absence of the *de novo* methyltransferases 3a and 3b, as expected (S2 Fig b-i, S3 Table); RCP values increased as expected for several, though not all, loci. Results for cells that lacked only one of the two DNA methyltransferases were even more variable across loci (S2 Fig, S3 Table). In some cases, loss of a single Dnmt3 enzyme had the predicted impact of increasing RCP (*L1* in 3a KO, $p < 10^{-16}$, S2 Fig h; *L1* in 3b KO, $p = 0.002$, S2 Fig h; *IAP* in 3a KO, $p = 0.002$, S2 Fig g; *IAP* in 3b KO, $p = 0.029$, S2 Fig g; two-tailed PTs), as was observed for *Lep* in our data. For one locus, *B1*, knockout of both of the Dnmt3s resulted in significantly increased RCP ($p < 10^{-16}$, two-tailed PT), though neither of the single knockouts did (S2 Fig f). Most surprisingly, for two loci, loss of either of the Dnmt3s decreased RCP (*Igf2*, $p < 0.016$, S2 Fig c; *Igf2*, $p < 10^{-16}$, S2 Fig c; two-tailed PTs). These counterintuitive results likely reflect variation across loci in the roles of the individual DNA methlytransferases — and possibly the demethylation machinery — in shaping overall methylation levels for various loci and categories of genic elements [56].

References

56. Baubec T, Colombo DF, Wirbelauer C, Schmidt J, Burger L, Krebs AR, et al. Genomic profiling of DNA methyltransferases reveals a role for DNMT3B in genic methylation. Nature. 2015;520(7546):243–247.
